# Perfluorooctanoic Acid Exposure Causes Macrophage Ammonia Retention and Induces Spontaneous Miscarriages

**DOI:** 10.1002/advs.202506994

**Published:** 2026-01-04

**Authors:** Yongbo Zhao, Yijun Zhang, Hanyu Rao, Jiani Sun, Zhiyi Pan, Liping Jin, Yan Zhao

**Affiliations:** ^1^ Obstetrics & Gynecology Hospital of Fudan University Shanghai Key Lab of Reproduction and Development Shanghai Key Lab of Female Reproductive Endocrine Related Diseases Shanghai China; ^2^ Shanghai Key Laboratory of Maternal Fetal Medicine, Shanghai Institute of Maternal‐Fetal Medicine and Gynecologic Oncology, Shanghai First Maternity and Infant Hospital School of Medicine, Tongji University Shanghai 200092 China; ^3^ The Third Affiliated Hospital of Zhengzhou University Zhengzhou China

**Keywords:** ammonia retention, macrophage, perfluorooctanoic acid, spontaneous miscarriage

## Abstract

Spontaneous miscarriage, the most prevalent complication of early pregnancy, poses substantial risks to maternal health worldwide. Perfluorooctanoic acid (PFOA) is a ubiquitous environmental persistent organic pollutant. Human epidemiological studies have linked PFOA exposure to spontaneous miscarriages, yet the underlying mechanisms have been rarely explored. In this study, we found PFOA exposure induced embryonic absorption in pregnant mice by causing ammonia retention in macrophages. Excessive ammonia disrupted mitochondrial function and compromised lysosomal integrity, which ultimately impaired macrophage function. Furthermore, lysosomal dysfunction reduced secretion of cathepsin B (CTSB) and led to decreased macrophage infiltration and diminished trophoblast invasion. Mechanistically, PFOA exposure led to macrophages ammonia retention by promoting the glutaminolysis through the upregulation of glutaminase (GLS) expression. By downregulating the inhibitor of DNA binding protein 3 (ID3), PFOA enhanced nuclear translocation and DNA‐binding affinity of transcription factor 12 (TCF12), which directly activated glutaminase (GLS) transcription to fuel glutamine catabolism. Collectively, our findings delineated a previously unrecognized pathway linking environmental PFOA exposure to spontaneous miscarriage via ammonia‐driven macrophage impairment.

## Graphical abstract

PFOA exposure induces pregnancy loss by promoting glutaminolysis, which further causes ammonia accumulation in macrophages. Cellular ammonia retention results in damage to mitochondria and lysosomes, which leads to cell death eventually. Impaired lysosomes also decrease the secretion of the Cathepsin B (CTSB), and attenuate macrophage infiltration and trophoblast invasion.

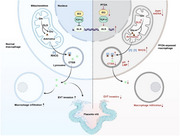

## 1 Introduction

Spontaneous miscarriages, a prevalent early pregnancy complication, impacts approximately 15% of clinically recognized pregnancies [1]. It not only poses a serious threat to women's reproductive health but also imposes tremendous psychological burdens and mental distress on women and their families [2]. The known factors that contribute to spontaneous miscarriages include embryonic chromosol abnormalities, anatomical abnormalities of the female reproductive tract, and maternal endocrine or immune disorders [3]. However, the etiology of spontaneous miscarriages cannot be fully explained by these factors, with approximately 50% of cases remaining idiopathic [1]. Recently, human epidemiological studies have suggested that environmental persistent organic pollutants (POPs) exposure may also play a role in the development of spontaneous miscarriages [4, 5, 6].

Per‐ and polyfluoroalkyl substances (PFAS) are a large group of synthetic chemicals that may cause health risks to human. The common PFAS include perfluorooctanoic acid (PFOA), perfluorooctane sulphonate (PFOS), perfluorononanoic acid (PFNA), perfluorodecanoic acid (PFDA), and perfluorohexanesulfonic acid (PFHxS) [7, 8]. As the most prevalent and persistent homologues within the PFAS family, PFOA has been widely used in numerous industrial applications and consumer goods, such as polymerization aid, industrial detergent or fire‐fighting foams [9]. Human exposure to PFOA can occur through inhalation, ingestion, or dermal contact, and notable human burdens have been documented [10]. PFOA is recognized as an endocrine disruptor and has demonstrated reproductive toxicity in animal studies [11, 12]. Recent epidemiological studies suggest that prenatal exposure to PFOA is associated with an increased risk of spontaneous miscarriage. In a case‐control study, a monotonic increase in miscarriage odds with increased serum PFOA levels was observed, showing an odds ratio (OR) of 2.2 (95% CI: 1.2 ‐ 3.9) for the highest vs the lowest PFOA quartile [13]. Supporting this, an analysis of the prospective Swedish SELMA cohort reported an OR of 1.48 (95% CI: 1.09 ‐ 2.01) for miscarriage in relation to higher PFOA levels, after adjusting for key confounders such as parity, age, and smoking [14]. These findings are consistent with several other studies reporting the positive relationship between PFOA levels and elevated miscarriage incidence [15, 16, 17]. Despite this accumulating epidemiological evidence, the underlying biological mechanisms by which PFOA exposure leads to miscarriage remain poorly understood.

The immune regulation at the maternal‐fetal interface played a pivotal role in establishing and maintaining a healthy pregnancy [18]. Decidual macrophages, which make up approximately 10 ‐ 20% of decidual immune cells, are key components of the immune tolerance network at this interface. They participate in critical processes necessary for pregnancy support, including tissue remodeling, immune tolerance, and the maintenance of immune balance [19]. Dysfunction of macrophages can disrupt these processes, potentially leading to pregnancy loss [20, 21]. A recent study has indicated that exposure to PFOA may decrease cell viability and increase apoptosis in macrophages [22]. However, the signaling pathway and regulatory mechanism were rarely investigated.

Glutamine metabolism is a crucial pathway that supports cellular energy generation and biosynthesis, during which ammonia is yielded as a byproduct [23]. Excessive ammonia accumulation can disrupt cellular function and induce cell death, particularly in immune cells. This distinct form of cell death, characterized by lysosomal alkalization and mitochondrial swelling, was first described by Zhang et al. [24]. Emerging evidence suggests that PFOA may exert its toxic effects by altering metabolic pathways [22, 25, 26]. A recent study has indicated that PFOA exposure can increase glutamine consumption in human intestinal cells [27]. However, whether exposure to PFOA disrupts glutamine metabolism, leads to ammonia accumulation, and consequently impairs macrophage function should be further explored.

In this study, we demonstrate that exposure to PFOA induces spontaneous miscarriages by triggering ammonia‐related cell death in macrophages. Through an in‐depth exploration of the molecular and cellular mechanisms underpinning this process, we aim to offer novel insights into the reproductive toxicity of PFOA.

## 2 Results

### 2.1 PFOA Exposure Induces Pregnancy Loss

**FIGURE 1:**
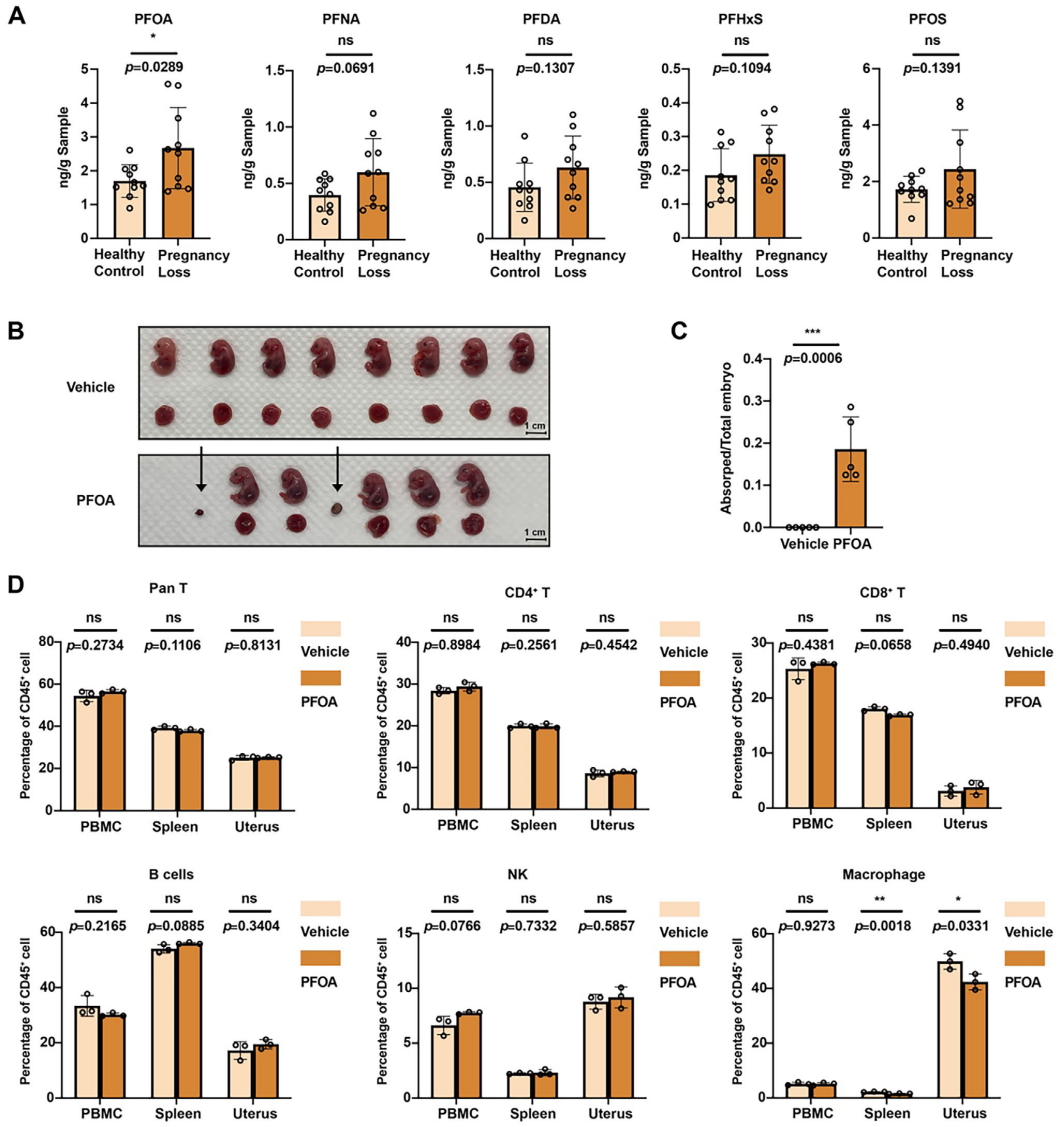
PFOA exposure induces embryo absorption and decreases the percentage of macrophages. A) The concentrations of 5 PFAS in the decidua of healthy controls (n = 10) and patients with spontaneous miscarriages (n = 10). B) A representative photograph of embryos and placentas from pregnant mice exposed to vehicle or PFOA (arrows indicate embryo absorption site, scale bar = 1 cm). C) Statistical analysis of the absorption rate of pregnant mice exposed to vehicle or PFOA (n = 5). D) Percentages of various immune cells in the peripheral blood, spleen, and uterus of pregnant mice exposed to vehicle or PFOA (n = 3). Data were presented as mean ± SEM and analyzed with two‐tailed unpaired Student's *t*‐test. *p* < 0.05, ^*^; *p* < 0.01, ^**^; *p* < 0.001, ^***^; *p* < 0.0001, ^****^; no significance, ns.

Concentrations of 5 PFAS congers were measured in 20 human decidual tissue samples. As shown in Table S1, all decidual tissue samples had quantifiable levels of PFAS congers. Analyses of potential differences in PFAS concentrations between spontaneous miscarriages cases and healthy controls showed that only PFOA concentration was significantly higher in spontaneous miscarriages cases than those in healthy controls (Figure 1A). These results suggested that PFAS could reach the maternal‐fetal interface, and PFOA exposure was associated with spontaneous miscarriages.

Then, a PFOA‐exposed pregnant mouse model was conducted to confirm the observed associations between PFOA exposure and spontaneous miscarriages in human studies. Pregnant mice were exposed to 1 mg/kg/day of PFOA by gavages from gestational D 0.5 to D 14.5. Results showed that gestational PFOA exposure significantly induced embryonic absorption (Figure 1B,C) in pregnant mice. These results confirmed the embryonic developmental toxicity in rodents.

### 2.2 PFOA Exposure Decreases the Percentage of Macrophages in Spleen and Uterus

Gestation presents a significant immunological challenge to the maternal immune system, as the fetus can be considered a semi‐allogeneic ‘allograft’ requiring complex immunomodulatory mechanisms for tolerance [28]. Then, the effects of PFOA exposure on heterogeneous immune cell subsets in the peripheral blood, spleen, and uterus of pregnant mice were assessed. As shown in Figure 1D, PFOA exposure decreased the percentage of macrophages in the spleen and uterus. However, there was no significant difference in the percentage of pan T, CD4^+^ T, CD8^+^ T, B cells, and NK cells between PFOA‐exposed mice and control mice.

### 2.3 PFOA Induces Pathological Ammonia Retention in Macrophages by Enhancing GLS‐mediated Glutamine Catabolism

**FIGURE 2:**
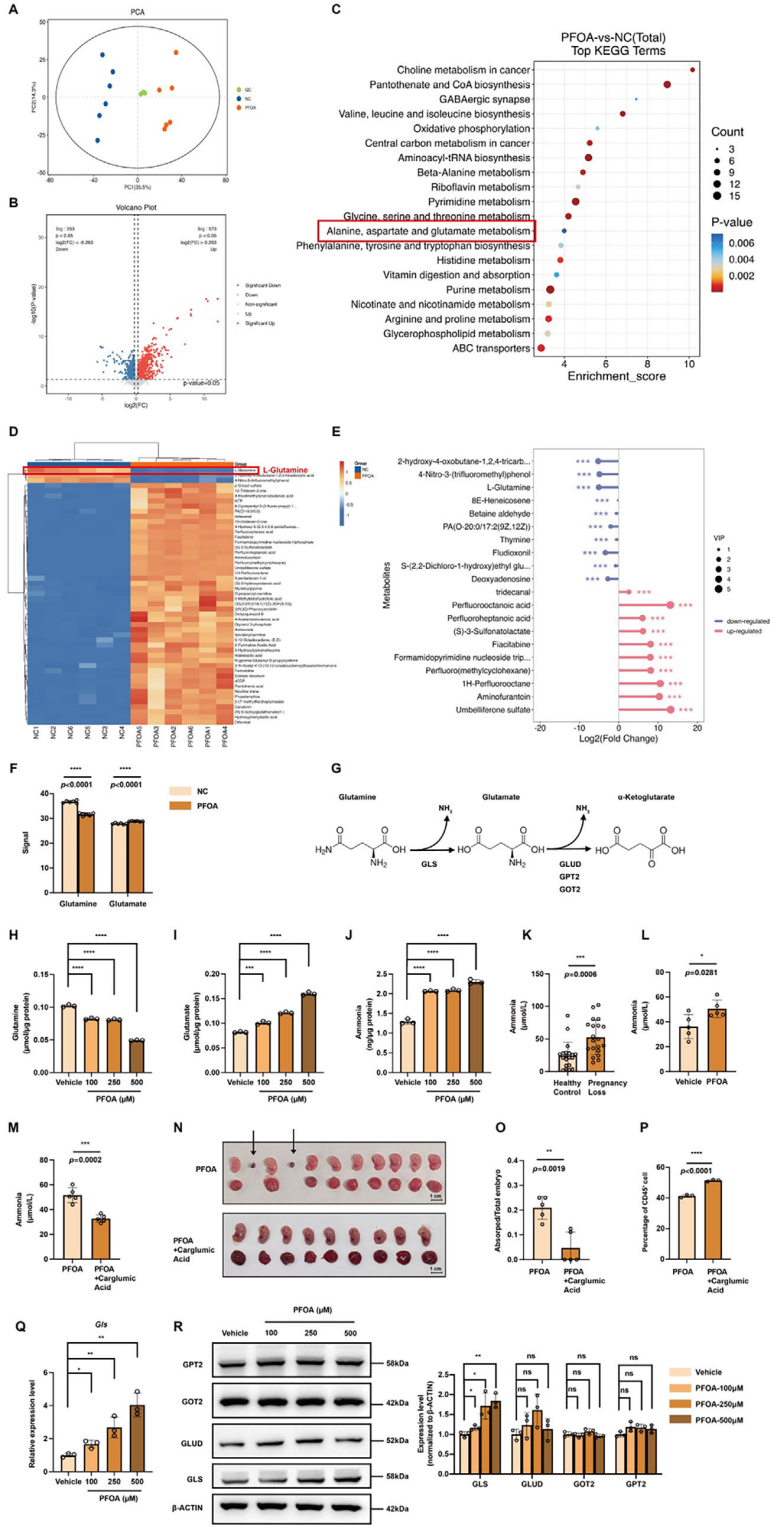
PFOA induces pathological ammonia retention in macrophages by enhancing GLS‐mediated glutamine catabolism. A) The PCA plot shows the clustering of specimens for untargeted metabolomics. Specimens include iBMDMs treated with 250 µM of PFOA (n = 5) and the vehicle group (n = 5). (B to D) The volcano plot, KEGG enrichment analysis, and heatmap of the differentially abundant metabolites. E) The top 10 significantly regulated metabolites in macrophages after PFOA exposure. F) The detected signal of glutamine and glutamate in untargeted metabolomics. G) A summary of the key process in glutaminolysis. H to J) Cellular concentration of glutamine, glutamate, and ammonia in macrophages treated with 0, 100, 250, and 500 µM of PFOA (n = 3). (K) Blood ammonia level in healthy controls (n = 20) and patients with spontaneous miscarriages (n = 20). (L) Blood ammonia level in control (n = 5) and PFOA‐exposed mice (n = 5). M) Mouse blood ammonia level of PFOA‐exposed group (n = 5) and PFOA + carglumic acid group (n = 5). N to O) A representative photograph of embryos and placentas from pregnant mice exposed to PFOA and PFOA + carglumic acid (arrows indicate embryo absorption site, scale bar = 1 cm) and statistical analysis of the absorption rate (n = 5). P) Percentages of macrophages gated from CD45^+^ cells in the uterus of pregnant mice in the PFOA‐exposed group and PFOA + carglumic acid group (n = 3). Q) mRNA expression level of *Gls* in macrophages treated with 0, 100, 250, and 500 µM of PFOA. R) Western blots of GPT2, GOT2, GLUD, and GLS in macrophages treated with 0, 100, 250, and 500 µM of PFOA (n = 3). The relative protein expression levels were normalized to β‐ACTIN expression. Data were presented as mean ± SEM and analyzed with two‐tailed unpaired Student's *t*‐test or one‐way ANOVA. *p* < 0.05, ^*^; *p* < 0.01, ^**^; *p* < 0.001, ^***^; *p* < 0.0001, ^****^; no significance, ns.

To elucidate the mechanism underlying PFOA‐induced macrophage reduction at the maternal‐fetal interface, we performed an untargeted metabolomic profiling (Figure 2A). PFOA‐exposed macrophages exhibited 573 upregulated and 353 downregulated metabolites (Figure 2B). KEGG analysis highlighted glutamine metabolism as one of the most perturbed pathways (Figure 2C). Specifically, L‐glutamine levels markedly decreased following PFOA treatment, while its metabolic derivative, glutamate, exhibited a slight increase (Figure 2D–F). The mitochondrial glutaminolysis cascade initiates with glutaminase (GLS)‐mediated conversion of glutamine to glutamate, followed by glutamate dehydrogenase (GLUD)/ glutamate oxaloacetate transaminase 2 (GOT2)/ glutamate pyruvate transaminase 2 (GPT2)‐driven transformation to α‐KG with concomitant ammonia release (Figure 2G). Consistently, PFOA exposure induced glutamine reduction with concomitant glutamate/ammonia accumulation in macrophages (Figure 2H–J). Besides, patients with spontaneous miscarriages or PFOA‐exposed pregnant mice also demonstrated hyperammonemia (Figure 2K,L). Previous studies have reported that hyperammonemia could cause immune dysfunction [29], as evidenced by impaired T cell proliferation and neutrophil phagocytosis [30, 31, 32]. Based on this association, we hypothesized that elevated ammonia levels might mediate the observed reduction in uterine macrophages following PFOA exposure. Carglumic acid, a carbamoyl phosphate synthetase I (CPS1) activator facilitating ammonia detoxification, could eliminate ammonia. Administration of carglumic acid to PFOA‐exposed mice normalized blood ammonia levels, attenuated fetal resorptions, and reversed the reduction in uterine macrophage infiltration (Figure 2M–P). These findings collectively suggested that ammonia overload was the key pathogenic mediator in PFOA‐induced pregnancy loss and uterine macrophage infiltration.

We postulated that ammonia accumulation might stem from augmented glutaminolytic enzyme activity. To verify this, we quantitatively assessed the expression profiles of glutaminolysis‐related enzymes (GLS, GLUD, GOT2, and GPT2) in PFOA‐treated macrophages. Strikingly, only GLS exhibited significant transcriptional upregulation, whereas other enzymes showed no significant alterations (Figure 2Q,R). These data mechanistically link PFOA‐induced GLS overexpression to enhanced glutaminolytic flux and consequent ammonia accumulation in macrophages.

### 2.4 PFOA Exposure Upregulates GLS Expression Through the ID3‐TCF12 Axis

**FIGURE 3:**
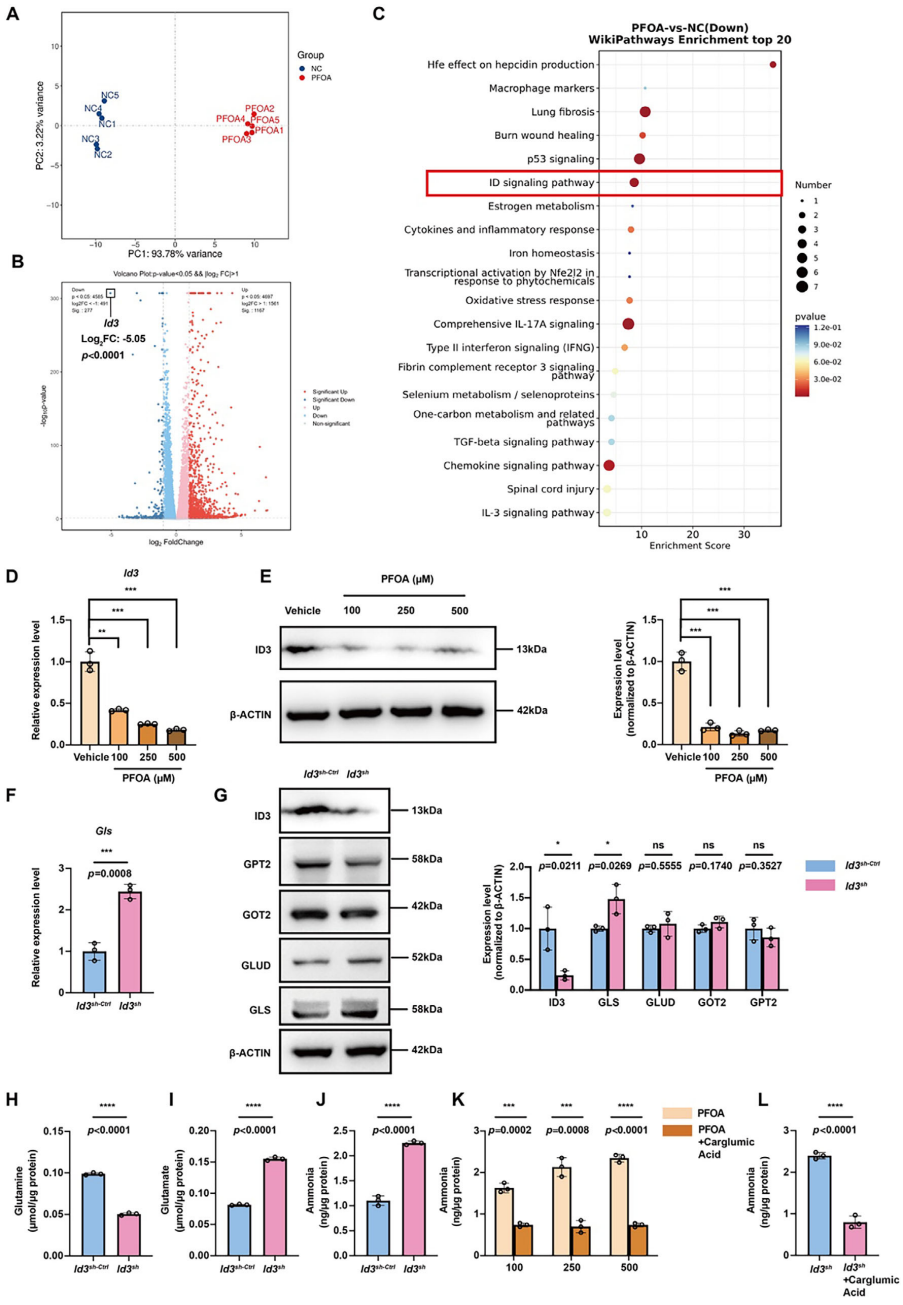
PFOA promotes GLS expression through down‐regulation of ID3. A) The PCA plot shows the clustering of specimens for RNA sequence. Specimens include iBMDMs treated with 250 µM PFOA (n = 5) and the vehicle group (n = 5). B,C) The volcano plot and KEGG enrichment analysis of the differentially expressed genes. D) mRNA expression level of *Id3* in macrophages treated with 0, 100, 250, and 500 µM of PFOA (n = 3). E) Western blots of ID3 in macrophages treated with 0, 100, 250, and 500 µM of PFOA (n = 3). The relative protein expression levels were normalized to β‐ACTIN expression. F) mRNA expression level of *Gls* in *Id3* ^sh‐Ctrl^ and *Id3* ^sh^ macrophages (n = 3). G) Western blots of ID3, GPT2, GOT2, GLUD, and GLS in *Id3* ^sh‐Ctrl^ and *Id3* ^sh^ macrophages (n = 3). The relative protein expression levels were normalized to β‐ACTIN expression. H to J) Cellular concentration of glutamine, glutamate, and ammonia in *Id3* ^sh‐Ctrl^ and *Id3* ^sh^ macrophages (n = 3). K) Cellular ammonia concentration in macrophages treated with PFOA and PFOA + carglumic acid (10 µM) (n = 3). L) Cellular ammonia concentration in *Id3* ^sh‐Ctrl^ and *Id3* ^sh^ macrophages (n = 3). Data were presented as mean ± SEM and analyzed with two‐tailed unpaired Student's *t*‐test or one‐way ANOVA. *p* < 0.05, ^*^; *p* < 0.01, ^**^; *p* < 0.001, ^***^; *p* < 0.0001, ^****^; no significance, ns.

To investigate the mechanistic basis of PFOA‐mediated GLS regulation, RNA‐seq analysis of PFOA‐exposed immortalized bone marrow‐derived macrophages (iBMDMs) revealed 1167 upregulated and 277 downregulated genes (Figure 3A,B). Notably, the inhibitor of DNA‐binding protein 3 (ID3) demonstrated the most marked downregulation (log2FC: ‐5.0487, *p* < 0.001), with KEGG analysis implicating ID signaling pathway disruption (Figure 3C). These findings suggest ID3 as a potential transcriptional regulator of GLS expression.

Consistent with our RNA‐seq data, PFOA exposure significantly suppressed both mRNA and protein expression of ID3 (Figure 3D,E). To mechanistically interrogate this observation, we generated stable ID3‐knockdown iBMDM cells through shRNA‐mediated silencing. Genetic ablation of ID3 selectively upregulated GLS while maintaining normal expression levels of GLUD, GOT2, and GPT2 (Figure 3F,G). Similar to PFOA exposure, ID3 knockdown led to decreased intracellular glutamine levels and increased accumulation of glutamate and ammonia (Figure 3H–J). And overexpression of ID3 can significantly increase GLS expression in PFOA‐treated cells (Figure S2A). ID3 overexpression alleviated PFOA‐induced glutamine depletion and glutamate/ammonia accumulation (Figure S2B–D). We also found that supplementation with carglumic acid effectively eliminated ammonia accumulation induced by both PFOA and ID3 knockdown (Figure 3K,L). These findings establish ID3 as a critical upstream transcriptional regulator of GLS in the cellular response to PFOA exposure.

To investigate the role of ID3 in PFOA‐induced pregnancy loss, we developed an ID3‐deficient murine model. *Id3* ^−/−^ pregnant mice exhibited significantly elevated embryonic resorption rates and systemic hyperammonemia (Figure S1A–C). Carglumic acid administration normalized both ammonia levels and pregnancy outcomes (Figure S1D,E). Bone marrow‐derived macrophages (BMDMs) from *Id3* ^−/−^ mice recapitulated the PFOA‐associated metabolic profile, showing depleted glutamine stores with concomitant glutamate/ammonia accumulation (Figure S1F–H). Comprehensive immune profiling revealed selective macrophage reduction in splenic and uterine tissues, indicating a critical regulatory role for ID3 in macrophage homeostasis (Figure S1I).

**FIGURE 4:**
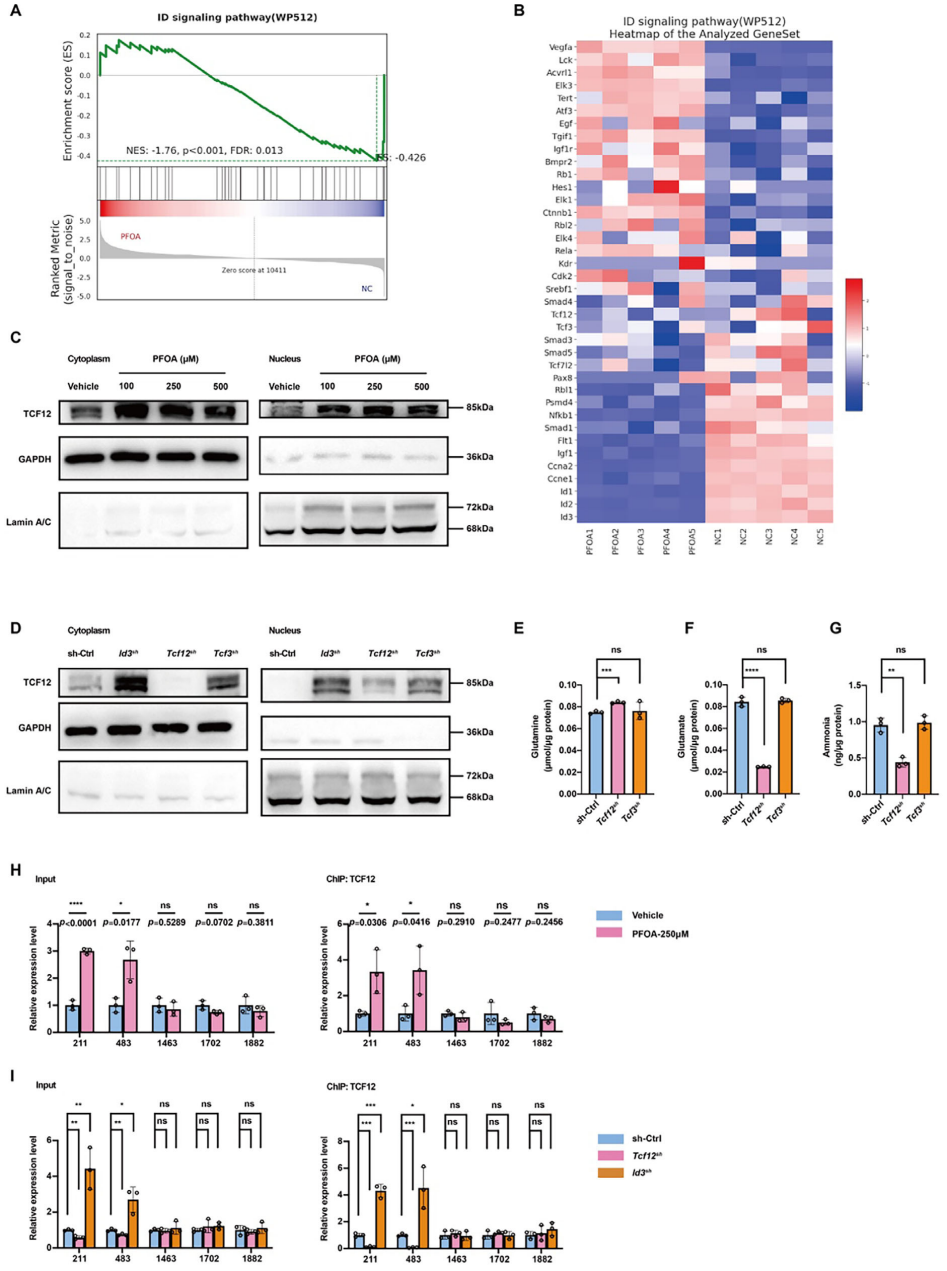
PFOA exposure upregulates GLS expression through the ID3‐TCF12 axis. A,B) GSEA enrichment plot and heatmap of the differentially expressed genes on the ID signaling pathway. C) Western blots of TCF12 in the cytoplasm and nucleus of macrophages treated with 0, 100, 250, and 500 µM of PFOA. D) Western blots of TCF12 in the cytoplasm and nucleus of sh‐Ctrl, *Id3* ^sh^, *Tcf12* ^sh^, and *Tcf3* ^sh^ macrophages. E to G) Cellular concentration of glutamine, glutamate, and ammonia in sh‐Ctrl, *Tcf12* ^sh^, and *Tcf3* ^sh^ macrophages (n = 3). (H) Using ChIP‐qPCR to determine the binding site of TCF12 on the promoter region of *Gls* in macrophages treated with 250 µm of PFOA (n = 3). I) Using ChIP‐qPCR to determine the binding site of TCF12 on the promoter region of *Gls* in sh‐Ctrl, *Tcf12* ^sh^, and *Id3* ^sh^ macrophages (n = 3). Data were presented as mean ± SEM and analyzed with two‐tailed unpaired Student's *t*‐test. *p* < 0.05, ^*^; *p* < 0.01, ^**^; *p* < 0.001, ^***^; *p* < 0.0001, ^****^; no significance, ns.

ID3, a class V helix‐loop‐helix (HLH) transcription factor lacking a DNA‐binding domain, negatively regulates gene expression through inactive complex formation with HLH‐containing partners. Our results showed that PFOA exposure disrupts ID signaling, significantly affecting TCF3 and TCF12 expression (Figure 4A,B; Table S2). While TCF3 is a known ID3 target [33], TCF12 is predicted to be able to interact with ID3 (Figure S3A). Both PFOA exposure and ID3 knockdown increased TCF12 expression and nuclear translocation (Figure 4C,D). TCF12 knockdown alone replicated PFOA‐induced metabolic changes in glutamine/glutamate metabolism and ammonia levels, whereas TCF3 knockdown did not (Figure 4E–G). This suggests TCF12 as the primary target of ID3. TCF12, predicted by the JASPAR database to have five potential binding sites in the *Gls* promoter (Figure S3B,C), was found to directly bind to two CAGTTG motifs in the *Gls* promoter under PFOA exposure or ID3 knockdown (Figure 4H,I). We propose that PFOA downregulates ID3, relieving its inhibition on TCF12 and facilitating the transcriptional activation of *Gls* through direct promoter binding.

### 2.5 Excessive Cellular Ammonia Decreases Macrophage Viability

**FIGURE 5:**
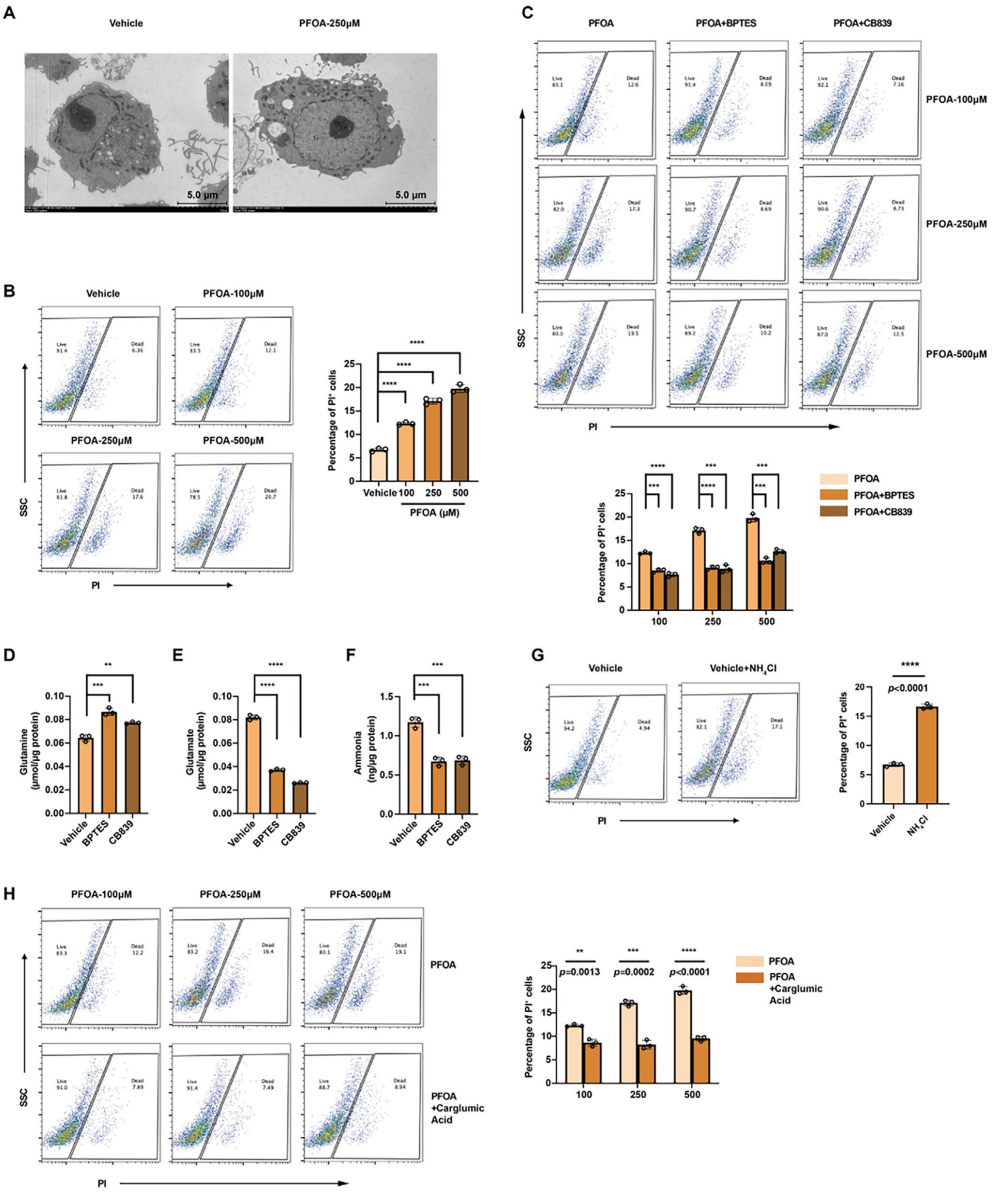
Excessive cellular ammonia decreases macrophage viability. A) Representative electron microscopy of macrophages treated with 0 and 250 µm of PFOA. Scale bar = 500 nm. B) PI staining of macrophages treated with 0, 100, 250, and 500 µM of PFOA (n = 3). C) PI staining of macrophages treated with PFOA, PFOA + BPTES, and PFOA + CB839 (n = 3). D–F) Cellular concentration of glutamine, glutamate, and ammonia in macrophages treated with BPTES (10 µM) and CB839 (5 µmM) (n = 3). G) PI staining of macrophages treated with NH_4_Cl (10 mM) (n = 3). H) PI staining of macrophages treated with PFOA and PFOA + carglumic acid (n = 3). Data were presented as mean ± SEM and analyzed with two‐tailed unpaired Student's *t*‐test or one‐way ANOVA. *p* < 0.05, ^*^; *p* < 0.01, ^**^; *p* < 0.001, ^***^; *p* < 0.0001, ^****^; no significance, ns.

As shown in Figure 5A and Figure S4A, PFOA exposure and ID3 knockdown both impaired macrophage viability. Morphologically, cytoplasmic vacuoles, a hallmark of ammonia‐induced cell death [24], were observed in PFOA‐treated and ID3‐knockdown macrophages (Figure 5A; Figure S4A). PI staining confirmed that PFOA exposure and ID3 knockdown significantly decreased macrophage viability (Figure 5B; Figure S4B). To confirm that GLS‐mediated glutaminolysis is the primary source of ammonia accumulation, we used GLS‐specific inhibitors BPTES and CB839 to conduct intervention research. Both BPTES and CB839 rescued PFOA‐ or ID3 knockdown‐induced macrophage cell death (Figure 5C; Figure S4C). Inhibiting GLS increased glutamine levels and reduced glutamate and ammonia (Figure 5D–F). Exogenous NH_4_Cl recapitulated this phenotype, further confirming the association between ammonia overload and macrophage cell death (Figure 5G). Additionally, carglumic acid treatment improved macrophage viability under these conditions (Figure 5H; Figure S4D). Overexpression of ID3 significantly rescued macrophage viability when treated with PFOA (Figure S4E). Collectively, these results indicate that cellular ammonia accumulation is responsible for macrophage cell death.

### 2.6 Excessive Cellular Ammonia Triggers Mitochondrial Dysfunction

**FIGURE 6:**
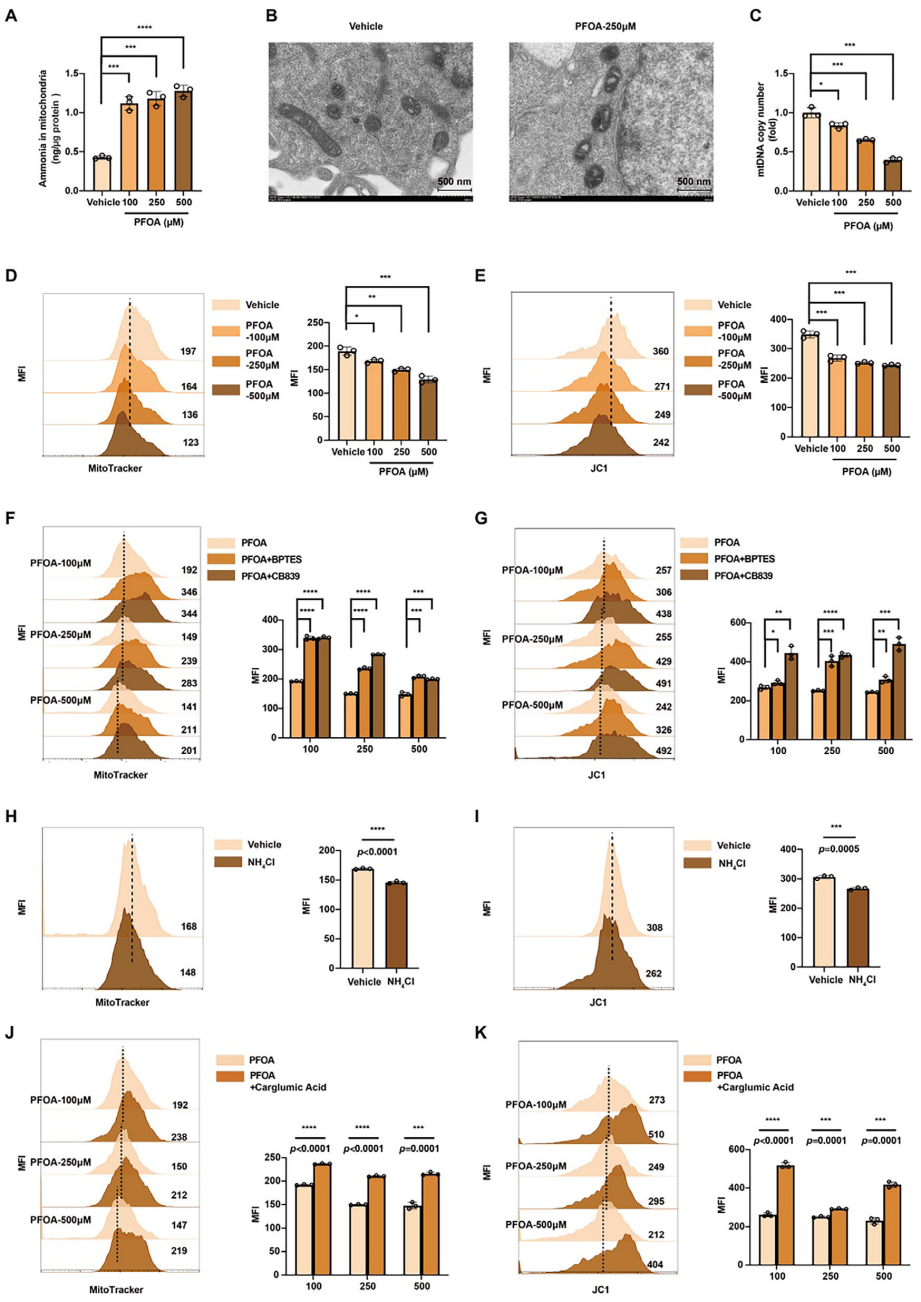
Excessive cellular ammonia triggers mitochondrial dysfunction. (A) Concentration of mitochondrial ammonia in macrophages treated with 0, 100, 250, and 500 µM of PFOA (n = 3). B) Representative electron microscopy of mitochondria in macrophages treated with 0 and 250 µM of PFOA. Scale bar = 500 nm. C) The copy numbers of mitochondrial DNA in macrophages treated with 0, 100, 250, and 500 µM of PFOA (n = 3). D,E) The MFI of Mito Tracker and JC‐1 in macrophages treated with 0, 100, 250, and 500 µM of PFOA (n = 3). F and G) The MFI of Mito Tracker and JC‐1 in macrophages treated with PFOA, PFOA + BPTES, and PFOA + CB839 (n = 3). H and I) The MFI of Mito Tracker and JC‐1 in macrophages treated with NH_4_Cl (n = 3). J and K) The MFI of Mito Tracker and JC‐1 in macrophages treated with PFOA and PFOA + carglumic acid (n = 3). Data were presented as mean ± SEM and analyzed with two‐tailed unpaired Student's *t*‐test or one‐way ANOVA. *p* < 0.05, ^*^; *p* < 0.01, ^**^; *p* < 0.001, ^***^; *p* < 0.0001, ^****^; no significance, ns.

We next explored how ammonia accumulation triggers macrophage death. Since glutaminolysis primarily occurs in mitochondria, ammonia retention might induce mitochondrial dysfunction. As a weak base, ammonia consumes protons to form ammonium, potentially disrupting the mitochondrial proton gradient and membrane potential (MMP) [34]. We found both PFOA exposure and ID3 knockdown significantly increased mitochondrial ammonia levels (Figure 6A; Figure S5A). Transmission electron microscopy (TEM) revealed severe ultrastructural abnormalities, including cristae fragmentation and matrix swelling in both PFOA‐treated and ID3‐deficient macrophages (Figure 6B; Figure S5B). Moreover, PFOA exposure demonstrated a dose‐dependent mitochondrial dysfunction, characterized by decreased mtDNA copy numbers (Figure 6C), reduced mitochondrial number (Figure 6D), and MMP depolarization (Figure 6E), which is parallel to that in ID3‐deficient macrophages (Figure S5C–E). Notably, pharmacological blockade of GLS using BPTES/CB839 restored mitochondrial integrity in both PFOA‐exposed and ID3‐deficient macrophages (Figure 6F,G; Figure S5F,G). Consistently, we found NH_4_Cl treatment reduced mitochondrial content and MMP (Figure 6H,I). Enhancement of ammonia detoxification via carglumic acid also reversed the MMP collapse and mitochondrial number reduction (Figure 6J,K; Figure S5H,I), confirming ammonia overload as the primary driver of mitochondrial failure. In addition, ID3 overexpression increased mitochondrial content and MMP in macrophages exposed to PFOA (Figure S5J,K), indicating that downregulation of ID3 contributes to mitochondrial damage caused by PFOA.

### 2.7 Excessive Cellular Ammonia Compromises Lysosomal Function

**FIGURE 7:**
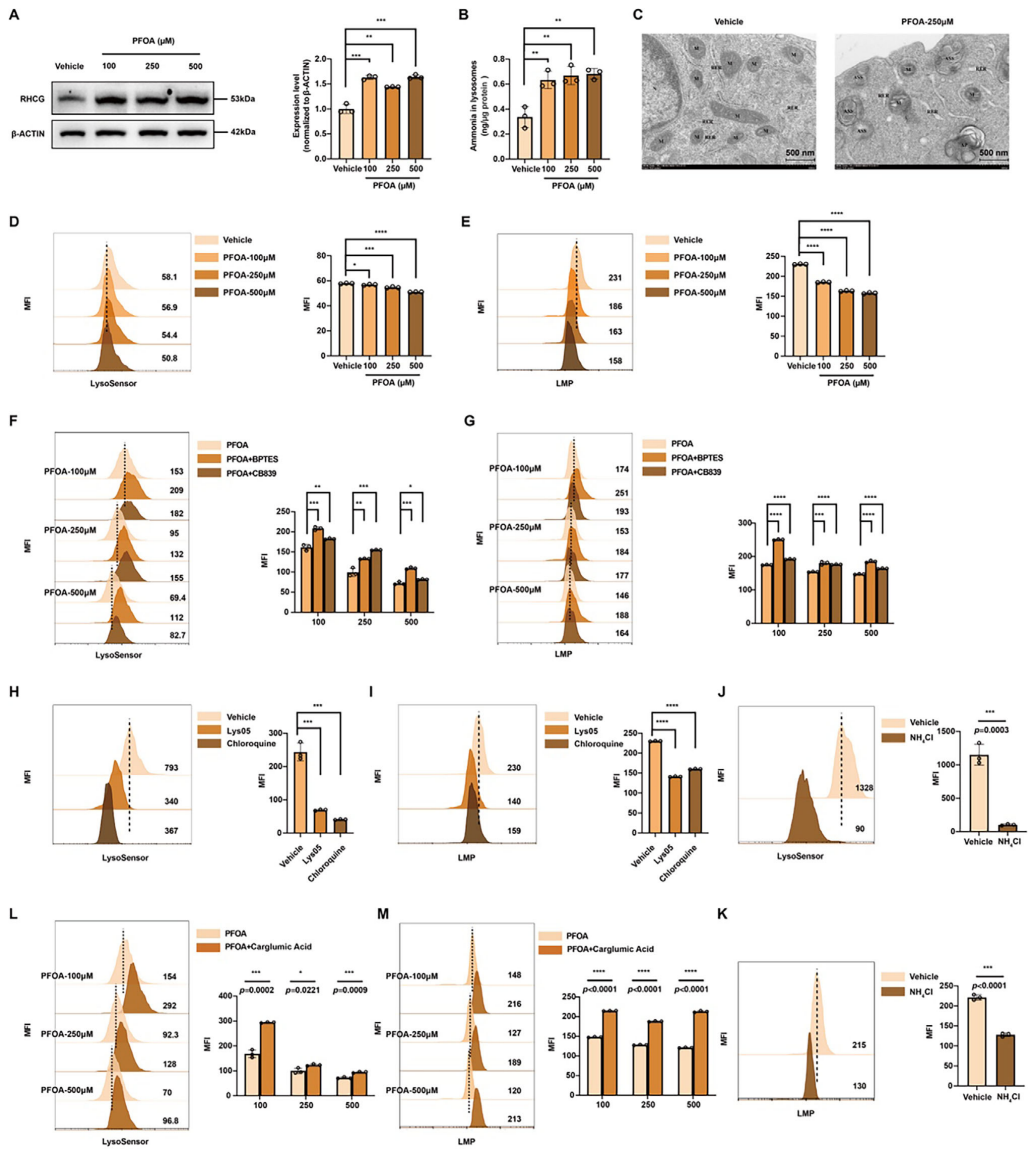
Excessive cellular ammonia compromises lysosomal function. A) Western blots of RHCG in macrophages treated with 0 and 250 µm of PFOA (n = 3). The relative protein expression levels were normalized to β‐ACTIN expression. B) Concentration of lysosomal ammonia in macrophages treated with 0, 100, 250, and 500 µM of PFOA (n = 3). C) Representative electron microscopy of macrophages treated with 0 and 250 µm of PFOA, mainly focused on autolysosomes. Scale bar = 500 nm. D and E) The MFI of LysoSensor and acridine orange (an indicator for LMP) in macrophages treated with 0, 100, 250, and 500 µm of PFOA (n = 3). F and G) The MFI of LysoSensor and acridine orange in macrophages treated with PFOA, PFOA + BPTES, and PFOA + CB839 (n = 3). H and I) The MFI of LysoSensor and acridine orange in macrophages treated with Lys05 or chloroquine (n = 3). J and K) The MFI of LysoSensor and acridine orange in macrophages treated with NH_4_Cl (n = 3). L and M) The MFI of LysoSensor and acridine orange in macrophages treated with PFOA and PFOA + carglumic acid (n = 3). Data were presented as mean ± SEM and analyzed with two‐tailed unpaired Student's *t*‐test or one‐way ANOVA. *p* < 0.05, ^*^; *p* < 0.01, ^**^; *p* < 0.001, ^***^; *p* < 0.0001, ^****^; no significance, ns.

Excess mitochondrial ammonia could diffuse directly through membranes or be passively transported as ammonium to other organelles [35]. As the primary acidic compartments, lysosomes serve as key sites for ammonium accumulation. Notably, the lysosomal ammonia transporter rhC glycoprotein (RHCG) [36] demonstrated significant upregulation in both PFOA‐exposed and ID3‐deficient macrophages, which correlated with increased lysosomal ammonia concentrations (Figure 7A,B; Figure S6A,B). TEM revealing autolysosome formation, and we observed lysosomal pH neutralization (quantified by LysoSensor) and increased membrane permeabilization (indicated by decreased MFI of acridine orange staining) in PFOA‐treated or *Id3* ^sh^ macrophages (Figure 7C–E; Figure S6C–E). Rescue experiments using glutaminase inhibitors (BPTES/CB839) reversed lysosomal damage (Figure 7F,G; Figure S6F,G), demonstrating mitochondrial‐derived ammonia as the pathogenic trigger for lysosomal dysfunction. Crucially, lysosomal V‐ATPase inhibitors (Lys05/chloroquine) mimicked these damaging effects, confirming pH disruption as the central mechanism (Figure 7H,I). NH_4_Cl treatment also led to lysosomal damage (Figure 7J,K), and ammonia scavenger (carglumic acid) reversed lysosomal damage in PFOA‐exposed or *Id3* ^sh^ macrophages (Figure 7L,M; Figure S6H,I), confirming the role of excessive cellular ammonia in lysosomal dysfunction. And PFOA induced lysosomal dysfunction was significantly rescued by overexpression of ID3 (Figure S6J,K), further indicating its important role in regulating cellular ammonia and lysosomal function.

### 2.8 Reduced Secretion of CTSB Leads to Decreased Macrophage Infiltration and Diminished Trophoblast Invasion

**FIGURE 8:**
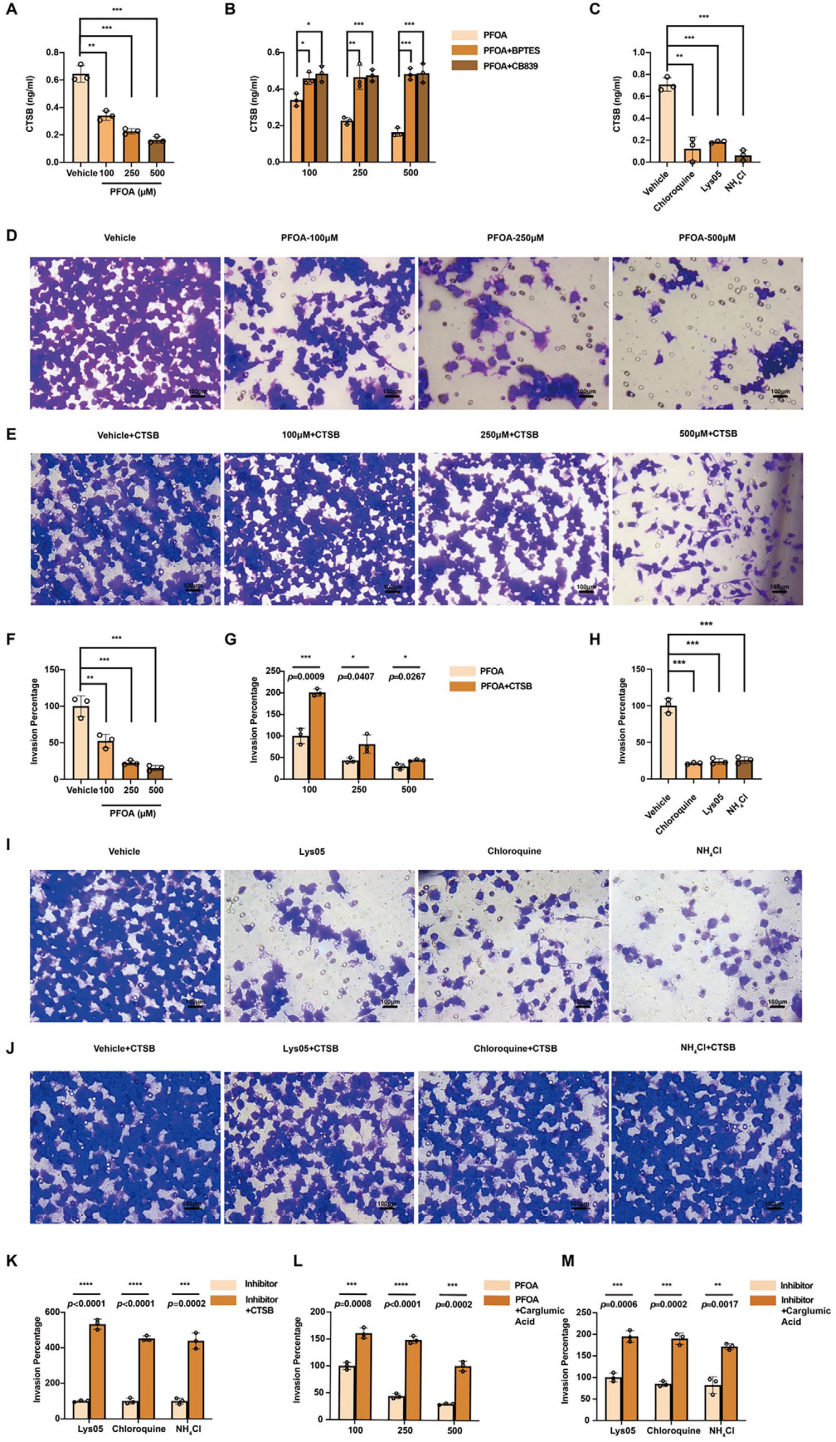
Reduced secretion of CTSB leads to decreased macrophage infiltration. (A) Concentration of CTSB in the culture supernatant of macrophages treated with 0, 100, 250, and 500 µM of PFOA (n = 3). B) Concentration of CTSB in the culture supernatant of macrophages treated with PFOA, PFOA + BPTES, and PFOA + CB839 (n = 3). C) Concentration of CTSB in the culture supernatant of macrophages treated with chloroquine, Lys05, or NH_4_Cl (n = 3). D) Representative images of macrophages treated with 0, 100, 250, and 500 µM of PFOA using transwell assay (scale bar = 100 µm). E) Representative images of macrophages treated with PFOA + CTSB (1 ng/ml) using transwell assay (scale bar = 100 µm). F) Statistical analysis of the infiltration ability of macrophages treated with 0, 100, 250, and 500 µM of PFOA (n = 3). G) Statistical analysis of the infiltration ability of macrophages treated with PFOA + CTSB (1 ng/mL) (n = 3). H) Statistical analysis of the infiltration ability of macrophages treated with chloroquine, Lys05, or NH_4_Cl (n = 3). I) Representative images of macrophages treated with lysosomal inhibitor (including Lys05, chloroquine, and NH_4_Cl) using transwell assay (scale bar = 100 µm). J) Representative images of macrophages treated with lysosomal inhibitor + CTSB using transwell assay (scale bar = 100 µm). K) Statistical analysis of the infiltration ability of macrophages treated with lysosomal inhibitor + CTSB (n = 3). L) Statistical analysis of the infiltration ability of macrophages treated with PFOA and PFOA + carglumic acid (n = 3). M) Statistical analysis of the infiltration ability of macrophages treated with lysosomal inhibitor and lysosomal inhibitor + carglumic acid group (n = 3). Data were presented as mean ± SEM and analyzed with two‐tailed unpaired Student's *t*‐test or one‐way ANOVA. *p* < 0.05, ^*^; *p* < 0.01, ^**^; *p* < 0.001, ^***^; *p* < 0.0001, ^****^; no significance, ns.

The above findings demonstrate that PFOA‐induced ammonia retention causes dual mitochondrial/lysosomal damage in macrophages, but its connection to pregnancy loss remained unclear. Cathepsin B (CTSB), a lysosomal protease, regulates extracellular matrix remodeling through secretion [37]. We found that CTSB cleavage and secretion were inhibited in PFOA‐exposed or ID3‐downregulated macrophages (Figure 8A; Figures S7A and S8A,B). GLS inhibitors restored CTSB secretion (Figure 8B; Figure S7B), while lysosomal V‐ATPase inhibitors or NH_4_Cl mimicked the effect, indicating lysosomal dysfunction reduced CTSB secretion (Figure 8C). Transwell assays showed that PFOA treatment or ID3 knockdown reduced macrophage infiltration, which was rescued by CTSB supplementation (Figure 8D–G; Figure S7C–E). Similarly, NH_4_Cl or inhibiting lysosomal activity with Lys05 or chloroquine reduced infiltration, which was reversed by exogenous CTSB (Figure 8H–K). Impaired infiltration caused by pharmacological or genetic interventions was also rescued by exogenous carglumic acid (Figure 8L,M; Figure S7F).

In addition to macrophage infiltration, trophoblast migration and invasion are essential for successful pregnancy [38]. As previous studies have shown that decidual‐infiltrated macrophages could regulate trophoblast activity through cellular crosstalk [39], we further explored whether macrophage‐derived CTSB could influence trophoblast invasion. As PFOA exposure can reduce the CTSB levels in the macrophage culture supernatant, this reduction also impaired trophoblast cell invasion in a dose‐dependent manner (Figure S8C,D). Exogenous CTSB alone can strongly promote HTR8 invasion (Figure S8E). In vivo, PFOA‐exposed mouse placentas showed lower CK7^+^ trophoblast invasion (Figure S8F). These results indicated that lysosomal damage in macrophages reduced CTSB secretion, affecting both macrophage infiltration and trophoblast invasion.

## 3 Discussion

PFOA is one of the most common POPs in the environment, and humans can be exposed to it through inhalation, ingestion, or dermal contact [40]. Elevated levels of PFOA have been reported in pregnant women, raising concerns about its potential effects on fetal growth and development [41, 42]. However, most epidemiological studies measured PFOA levels in blood or urine. Although they are the standard matrices for measuring systemic, body‐burden exposure, they may not accurately reflect the local accumulation in specific target organs or tissues [43]. The decidua is the core site for embryo implantation and development, as well as a direct target where environmental pollutants may disrupt pregnancy maintenance [44]. Direct measurement of PFOA in decidua allows a more precise assessment of its local burden and potential risks at the maternal‐fetal interface. In our study, we detected quantifiable levels of PFOA in human decidual tissue, indicating that PFOA can reach the maternal‐fetal interface and may interfere with the establishment and maintenance of pregnancy.

Moreover, we discovered that the decidual PFOA levels are significantly elevated among patients with spontaneous miscarriages compared to the healthy controls. Recently, some human epidemiological studies demonstrated that PFOA exposure was associated with increased risk of pregnancy loss [13, 15, 45, 46]. Supporting our findings, a Swedish prospective cohort study involving 1864 pregnant women [14] reported that higher PFOA exposure was associated with an increased risk of miscarriage, with a crude odds ratio (OR) of 1.38 (95% CI: 1.04 ‐ 1.83). After adjustment for covariates such as maternal age, parity, and tobacco smoke exposure during pregnancy, the association strengthened, yielding an adjusted OR of 1.48 (95% CI: 1.09 ‐ 2.01). These results are consistent with our own data and collectively reinforce the conclusion that PFOA exposure contributes to miscarriage risk.

In our in vivo study, we employed a dose of 1 mg/kg/day via oral gavage for 14 days to establish a PFOA‐exposed mouse model. Based on previous toxicological research, the commonly used PFOA doses range from 0.1 to as high as 40 mg/kg/day, with 1 mg/kg/day and 5 mg/kg/day being the most frequently applied [47, 48, 49, 50, 51, 52, 53, 54, 55, 56]. A clear dose‐response relationship was observed regarding the developmental toxicity [56]. Using a PFOA‐exposed pregnant mouse model, we confirmed that PFOA exposure induces reproductive toxicity in rodents.

Macrophages at the maternal‐fetal interface play critical roles in maintaining pregnancy, yet the immunotoxic effects of PFOA exposure on these cells have not been thoroughly investigated. One study reported that exposure to 200 µm PFOA significantly increased apoptosis and reduced cell viability in macrophage RAW264.7 cells [22]. In our study, we observed that macrophage proportion decreased specifically in the spleen and uterus, but not in the peripheral blood. This pattern suggests that PFOA exposure does not primarily act by reducing the circulating macrophage pool, but rather through impairing the tissue‐specific infiltration of macrophages at the maternal‐fetal interface. This finding highlights the potential role of PFOA in disrupting immune cell function during pregnancy.

Excessive ammonia accumulation is known to disrupt cellular function and induce cell death, particularly in immune cells [24, 57]. Glutaminolysis represents the primary source of ammonia production. Typically, ammonia is generated as a by‐product of amino acid catabolism [58], with glutamine and glutamate acting as the central hub of amino acid metabolism. Glutamine, as the most abundant circulating amino acid, serves as a primary nitrogen carrier [59, 60, 61] and undergoes rapid deamidation via glutaminase (GLS), a reaction that directly yields glutamate and ammonia. Supporting this, Zhang et al. revealed that glutamine acts as the main source of ammonia in CD8^+^ T cells by using nitrogen isotope tracing [24]. Similarly, we observed activated glutaminolysis and ammonia retention in macrophages.

Additionally, we demonstrated that PFOA exposure induced ammonia retention through the ID3‐TCF12‐GLS1 axis. ID3, a member of the HLH protein family [62], is critical for macrophage proliferation, differentiation, and polarization [63, 64, 65]. ID3 primarily exerts its effects by interacting with other HLH proteins, such as transcription factors from the TCF family, to inhibit their DNA‐binding activity [66]. While TCF3 is a well‐established target of ID3, our study revealed that TCF12, rather than TCF3, is activated to promote *Gls* transcription and ammonia retention. Although TCF12 is primarily recognized as an oncogene, its role in regulating glutamine metabolism provides new insights into its biological functions.

In humans, the primary pathway for ammonia detoxification is the urea cycle, which converts ammonia into nontoxic urea and occurs predominantly in hepatocytes and tumor cells [67, 68]. CPS1, the rate‐limiting enzyme in the urea cycle, directly incorporates ammonia into this pathway [69, 70]. Therefore, systemic administration of carglumic acid is expected to enhance urea cycle flux, primarily in hepatocytes where CPS1 is abundant, thereby contributing to the observed reduction in blood ammonia. Beyond this systemic effect, local ammonia balance within specific cellular microenvironments also holds physiological relevance. In our in vitro experiments, carglumic acid supplementation effectively alleviated ammonia accumulation, restored cell viability, and improved mitochondrial and lysosomal function in PFOA‐exposed macrophages. Collectively, these findings indicate that PFOA‑induced ammonia retention in macrophages contributes to spontaneous miscarriage, which can be significantly mitigated by carglumic acid.

The mechanism by which excessive ammonia causes cell death was also explored. As the primary acidic organelles in cells, lysosomes are likely targets of alkaline molecules such as ammonia. The ammonia transporter RHCG [36], initially identified in the distal tubules of mammalian kidneys, is expressed ubiquitously in multiple tissues [71]. Given the increase in lysosomal pH following PFOA exposure, we propose that lysosomes primarily take up ammonia rather than ammonium, as the former neutralizes acidity by consuming protons. Elevated lysosomal pH and lysosomal membrane permeability (LMP) can impair lysosomal enzyme activity. CTSB, a major lysosomal protease, is synthesized as an inactive pro‐enzyme and matures under acidic lysosomal conditions [72, 73]. The reduction in CTSB splicing and secretion observed in our study aligns with this mechanism.

CTSB plays dual roles in cellular homeostasis and extracellular interactions. It facilitates autophagy to eliminate misfolded proteins and is secreted to coordinate cell interactions [72]. Previous studies have shown that CTSB degrades the extracellular matrix and basement membrane, promoting tumor progression [37]. More recently, embryo‐derived CTSB was found to enhance decidualization and embryo implantation [15], suggesting its importance in pregnancy establishment and maintenance. In our study, we found that CTSB promotes macrophage infiltration, a process critical for spiral artery remodeling and immune tolerance during early pregnancy [21]. Additionally, macrophage‐derived CTSB facilitates trophoblast invasion, which is essential for placental development. Impaired trophoblast invasion is strongly associated with pregnancy complications such as miscarriages, preeclampsia, and fetal growth restriction [74]. Collectively, our findings suggest that reduced CTSB secretion may contribute to pregnancy loss by disrupting both macrophage function and trophoblast invasion.

In conclusion, our study elucidates a novel mechanism linking PFOA exposure to spontaneous miscarriage through ammonia‐driven macrophage cell death. By uncovering how PFOA disrupts macrophage function and ammonia metabolism at the maternal‐fetal interface, our findings provide critical insights into the reproductive toxicity of PFOA. These results not only deepen our understanding of the molecular pathways involved but also underscore the therapeutic potential of targeting ammonia metabolism to counteract PFOA's adverse effects. Given the persistent global public health concern posed by PFOA contamination, our research highlights the urgent need for stricter regulatory measures and effective public health interventions to reduce exposure and safeguard reproductive health.

## 4 Material and Methods

### 4.1 Chemicals and Reagents

PFOA (MACKLIN, #P815845) was dissolved in methanol and diluted to working concentrations of 100, 250, and 500 µm for in vitro experiments. For animal exposure, PFOA was administered at a dosage of 1 mg/kg/day. Carglumic acid (Selleck, #S5301) was dissolved in DMSO and diluted to a final concentration of 10 µM for cell treatment. In animal studies, carglumic acid was administered at 100 mg/kg/day. 10 mM NH_4_Cl (Sigma, #326372), 10 µm BPTES (Selleck, #S7753), 5 µm CB839 (Selleck, #S7655), 10 µM Lys05 (Selleck, #S8369), and 10 µM Chloroquine (Selleck, #S6999) were used for cell treatment in respective experiments. Recombinant human CTSB protein (Abclonal, #RP02824) and recombinant mouse CTSB protein (Abclonal, #RP02994) were reconstituted in PBS and diluted to 1 ng/mL for cell treatment.

### 4.2 Human Specimen

Decidual tissues and peripheral blood samples were obtained from both healthy pregnant women and patients experiencing spontaneous pregnancy loss at the Obstetrics and Gynecology Hospital of Fudan University from October 2024 to March 2025. All participants of the study provided a written form of consent. The study protocol was reviewed and approved by the hospital's Ethics Committee (kyy2024145) in accordance with the Declaration of Helsinki. Exclusion criteria included: 1) active infections; 2) severe endocrine or metabolic disorders such as PCOS, uncontrolled diabetes, or hypertension; 3) previous IVF treatment; 4) uterine anatomical abnormalities, including malformations, cervical insufficiency, or intrauterine adhesions; and 5) confirmed embryonic chromosomal abnormalities.

### 4.3 Decidual PFAS Measurement

Decidual tissue samples were homogenized in 1 mL of methanol for extraction. Following centrifugation, the supernatant was collected and mixed with the extraction buffer along with 200 mL of ultrapure water. The samples were then processed using HLB solid‐phase extraction columns that had been pre‐conditioned with 10 mL each of methanol and ultrapure water. The extracts were loaded onto the activated columns, washed with 4 mL of 25 mmol/L ammonium acetate solution, and subsequently eluted with sequential 5 mL aliquots of methanol and ethyl acetate. The eluates were concentrated to near‐dryness under a gentle nitrogen stream, reconstituted in 500 µL of methanol, filtered through a 0.22 µm membrane, and finally analyzed by LC‐MS/MS. All PFAS quantification procedures were performed with technical support from WEIPU Technology Co., Ltd. (Shanghai, China).

### 4.4 Mice

C57BL/6 wild‐type and *Id3* knockout mice were obtained from the Shanghai Laboratory Animal Research Center. Primers used for genotyping of *Id3* knockout mice were listed in Table S3. Mice aged 6 ‐ 8 weeks were housed under specific pathogen‐free conditions with controlled light, humidity, and temperature. All animals had ad libitum access to food and water. All experimental procedures were approved by the Institutional Animal Care and Use Committee of Fudan University (2024‐FCKYY‐166).

To establish the PFOA exposure model, 8‐week‐old female mice were paired with males overnight, and the presence of a vaginal plug marked gestational day 0.5 (GD0.5). Pregnant mice were then administered 1 mg/kg/day PFOA or vehicle control via daily oral gavage. The dosage was selected since it has been identified in previous dose‐response studies as the lowest effective dose for inducing relevant biological responses while remaining at or near the no‐observed‐adverse‐effect level (NOAEL) for key endpoints such as developmental toxicity [56]. It has also been set as the reference dose by the United States Environmental Protection Agency (EPA) for the Lifetime Health Advisory Level of 70 ppt PFOA for Drinking Water [55]. On GD14.5, mice were sacrificed for analysis. The embryo absorption rate was calculated using the formula R/(R+V), where R is the number of absorbed fetuses and V is the number of viable fetuses. Peripheral blood, spleen, and uterine tissues were collected for further investigation. Each group in the animal studies consisted of 5 pregnant mice, with an average of 6 ‐ 10 embryos collected per dam.

### 4.5 Cell Lines

The mouse immortalized bone marrow‐derived macrophage (iBMDM) was maintained in Dulbecco's Modified Eagle Medium (DMEM, VivaCell, #C3113‐0500), while the human monocyte cell line THP‐1 and trophoblast cell line HTR‐8/SVneo were cultured in RPMI 1640 medium (VivaCell, #C3010‐0500). All culture media were supplemented with 10% fetal bovine serum and 1% penicillin/streptomycin. Cells were maintained at 37°C in a humidified atmosphere containing 5% CO_2_. Regular mycoplasma testing was performed every three months to ensure all cell lines remained contamination‐free.

### 4.6 RNA Sequencing

Total RNA was isolated from cells using RNAiso Plus reagent (TaKaRa, #9109). RNA purity and concentration were determined by NanoDrop 2000 spectrophotometer (Thermo Scientific). RNA integrity was verified using the Agilent 2100 Bioanalyzer (Agilent Technologies). RNA‐seq libraries were prepared following the manufacturer's protocol using VAHTS Universal V6 RNA‐seq Library Prep Kit. The transcriptome sequencing and analysis were technically supported by OE Biotech Co., Ltd. (Shanghai, China).

The libraries were sequenced on an Illumina Novaseq 6000 platform to generate 150 bp paired‐end reads. After quality control, sequences were aligned to the mouse reference genome using HISAT2. Gene expression levels were quantified as FPKM values, and raw read counts were obtained using HTSeq‐count. Principal component analysis (PCA) was conducted in R (v3.2.0) to assess sample reproducibility. Differential gene expression analysis was performed with DESeq2, with significantly differentially expressed genes (DEGs) defined as those meeting the thresholds of *q*‐value < 0.05 and fold change > 1. Functional enrichment analysis of DEGs and Gene Set Enrichment Analysis (GSEA) were performed using R and GSEA software, respectively.

### 4.7 Metabolomics

For metabolite extraction, cell samples were treated with an ice‐cold methanol‐acetonitrile solution (2:1, v/v), ultrasonicated for 10 min in an ice‐water bath, and incubated at ‐20°C for 30 min. Following centrifugation at 13 000 rpm for 10 min, the supernatants were lyophilized and subsequently reconstituted in a methanol‐water mixture (1:4, v/v). After repeated centrifugation, the extracts were carefully collected using glass syringes, filtered through 0.22 µm membranes, and stored in LC vials at ‐80°C prior to analysis.

Untargeted metabolomic profiling was conducted by Shanghai Luming Biological Technology using an ACQUITY UPLC I‐Class Plus system (Waters) coupled to a Q‐Exactive mass spectrometer (Thermo Fisher Scientific) operating in both positive and negative ESI modes. Raw data were processed with Progenesis QI software (v2.3), followed by multivariate statistical analysis including OPLS‐DA and PLS‐DA to discriminate group‐specific metabolic patterns. Significant metabolites were identified based on Variable Importance of Projection (VIP) scores > 1.0 from OPLS‐DA models and *p*‐values < 0.05 from two‐tailed Student's *t*‐tests.

### 4.8 Cell Isolation

Mouse peripheral blood mononuclear cells (PBMCs) were isolated from orbital venous plexus blood collected in EDTA‐coated tubes following euthanasia. The blood was carefully layered onto Ficoll density gradient medium (Solarbio, #P8900) and centrifuged at 400 g for 30 min at room temperature. The PBMC interface was collected, washed with PBS, and resuspended in FACS staining buffer for subsequent analysis.

For splenocyte isolation, harvested spleens were mechanically dissociated using the flat end of a syringe plunger, filtered through a 70 µm cell strainer, and treated with RBC lysis buffer for 3 min at room temperature. After centrifugation at 1500 rpm for 3 min, the pelleted splenocytes were resuspended in FACS staining buffer.

Uterine cell isolation was performed by mincing uterine tissue from pregnant mice into small fragments, followed by enzymatic digestion with 1 mg/mL type IV collagenase (Sigma, #C5138) and 0.1 mg/mL DNase I (Sigma, #DN25) at 37°C with constant shaking at 220 rpm for 30 min. The digested suspension was filtered through a 70 µm strainer, centrifuged at 1500 rpm for 3 min, and the resulting cell pellet was resuspended in FACS staining buffer for flow cytometric analysis.

### 4.9 Flow Cytometry

For cell surface marker staining, single cell suspensions were blocked with anti‐CD16/32 (BD Biosciences, #553141) and then incubated with corresponding fluorescent‐labeled antibodies at 4°C in the dark for 30 min. All flow cytometric data were acquired with the BD Canto II instrument and analyzed with FlowJo software. The antibodies used in the flow cytometric assay are listed in Table S4.

### 4.10 Vector Construction and Transfection

To overexpress ID3 in macrophages, we first constructed a transient expression plasmid by cloning the coding sequence of mouse *Id3* and inserting it into the vector pCMV6‐AN‐DDK. Macrophages were seeded in 6‐well plates and grown to 50 ‐ 60% of confluence. For each well, 2.5 µg of ID3‐expressing plasmid or empty vector control was mixed with 5 µL of polyethyleneimine in the medium according to the manufacturer's protocol. The DNA‐lipid complexes were added to cells and incubated for 48 h before harvesting for experiments.

For lentivirus‐mediated gene knockdown, targeted shRNA sequences (Table S5) were cloned into the pLKO.1 vector. Lentiviral particles were produced by co‐transfecting HEK‐293T cells in 6 cm dishes with 4 µg pLKO.1‐shRNA, 3 µg psPAX2, and 1 µg pMD2.G using polyethyleneimine transfection reagent. Subsequently, iBMDMs were infected with the harvested lentivirus in medium containing 8 µg/ml polybrene (Solarbio, #H8761). Following 24 h of infection, stable knockdown cells were selected by treatment with 3 µg/mL puromycin (MCE, #HY‐B1743A) for 3 days. The knockdown efficiency of each gene was confirmed by western blot analysis of target protein expression levels.

### 4.11 Immunoblotting

Protein extraction was performed using RIPA lysis buffer (Epizyme, #PC101) containing protease and phosphatase inhibitors (NCM biotech, #P002). Following quantification with a BCA assay kit (Beyotime, #P0010), protein samples were denatured in 5 × loading buffer (NCM biotech, #WB2001) at 100°C for 10 min. The denatured proteins were then separated by sodium dodecyl sulfate‐polyacrylamide (SDS‐PAGE) gel electrophoresis and transferred onto nitrocellulose membranes (Cytiva, #10600001). After blocking with 5% non‐fat milk for 1 h at room temperature, the membranes were incubated with primary antibodies (listed in Table S6) overnight at 4°C. Following three washes with TBST buffer, the membranes were probed with HRP‐conjugated secondary antibodies (Epizyme, #LF102) for 1 h at room temperature. Protein bands were detected using the Tanon 4600 Chemiluminescent Imaging System, and relative protein expression levels were quantified by ImageJ software with β‐ACTIN serving as the loading control.

### 4.12 Quantitative RT‐PCR

Total RNA was isolated from cells using RNAiso Plus reagent (Takara, #C9109) and subsequently reverse transcribed into cDNA using PrimeScript RT Master Mix (Takara, #RR036). Quantitative real‐time PCR was performed on a QuantStudio system (Thermo Fisher/ABI) with SYBR Green detection reagent (Takara, #RR820A). The thermal cycling conditions were set as follows: initial denaturation at 95°C for 30 s, followed by 40 cycles of denaturation at 95°C for 5 s and combined annealing/ extension at 60°C for 34 s. Melting curve analysis was subsequently conducted from 60 to 95°C with a continuous temperature increment of 0.075°C/s to verify primer specificity. Gene expression levels were normalized to the housekeeping gene ACTB/Actb and calculated using the 2^–ΔΔCt^ method. All primers were synthesized by Biosune Biological Technology Co., LTD (Shanghai, China), and the primer sequences are listed in Table S7.

### 4.13 Immunohistochemistry Staining

Mouse placentas were fixed in 4% paraformaldehyde, embedded in paraffin, and sectioned at 4 µm thickness. Paraffin sections were technically supported by Wuhan Servicebio Technology Co., Ltd. (Wuhan, China). Following deparaffinization and rehydration, antigen retrieval was performed before blocking endogenous peroxidase activity. Tissue sections were then incubated with anti‐Cytokeratin 7 primary antibody (Servicebio, #GB12225) at 4°C overnight. After washing with PBST, sections were treated with HRP‐conjugated secondary antibody for 1 h at room temperature and developed with diaminobenzidine (DAB). Stained sections were imaged using a digital microscope.

Positive staining for CK7 was identified as moderate to strong intensity of cytoplasmic staining in cells with characteristic trophoblast morphology. The depth of invasion was quantified as the vertical distance from the basal chorionic plate to the deepest CK7‐positive area. 3 non‐overlapping fields along the axis of invasion were measured per placental section using ImageJ software, and the average of these triplicate measurements was taken as the final invasion depth for subsequent statistical analysis. To ensure reliability, all measurements were performed independently by two observers.

### 4.14 Measurements of Cellular Ammonia, Glutamine, and Glutamate

Cellular ammonia was measured using an ammonia/ammonium microplate assay kit (Absin, #abs580164‐96T). Cellular glutamine and glutamate were measured with a glutamine detection kit (Solarbio, #BC5305‐100T/48S) and a glutamate detection kit (Solarbio, #BC5215‐100T/48S), respectively. Briefly, cells from different treatment groups were collected and lysed on ice. One‐tenth of the cell lysate was used for protein quantification. The levels of ammonia, glutamine, and glutamate in whole cell lysate were measured according to the manufacturer's suggestions.

### 4.15 Propidium Iodide (PI) Staining

We measured cell viability by PI staining. Cells were harvested, centrifuged at 1200 rpm for 3 min, and incubated with PI working solution (Servicebio, #G1021) in the dark at room temperature for 20 min. Then, cells were centrifuged and resuspended in PBS. The MFI of stained cells was measured using flow cytometry.

### 4.16 Chromatin Immunoprecipitation Quantitative PCR (ChIP‐qPCR)

Chromatin immunoprecipitation (ChIP) assays were performed using the Sonication ChIP Kit (ABclonal, #RK20258) following the manufacturer's protocol. Cells were first crosslinked with 1% formaldehyde for 10 min at room temperature, followed by chromatin fragmentation via sonication to generate DNA fragments of 200 ‐ 700 bp. The sheared chromatin was then subjected to immunoprecipitation overnight at 4°C using either anti‐Tcf12 antibody (ABclonal, #A4146, 1:250) or control rabbit IgG (ABclonal, #RK20258, 1:250). Precipitated DNA fragments were subsequently analyzed by quantitative real‐time PCR with gene‐specific primers (Table S8) to determine target sequence enrichment.

### 4.17 Measurement of Mitochondrial Function

All experiments were performed according to the manufacturer's instructions. To specifically mark cellular mitochondria, cells were loaded with 200 nm Mito‐Tracker Green (Yeasen, #40742ES50) at 37°C for 30 min. To measure the mitochondrial membrane potential, cells were incubated with JC‐1 working solution (Beyotime, #C2006) at 37°C for 20 min. Flow cytometry was performed using a BD Canto II instrument, and the mean fluorescence intensity (MFI) of each channel was analyzed with FlowJo software. Mitochondrial DNA copy number was quantified by extracting total cellular DNA (TIANGEN, #DP304) followed by real‐time qPCR using mitochondrial‐specific primers (Table S7).

### 4.18 Measurement of Lysosomal Function

The lysosomal pH was measured using the lysosomal dye LysoSensor Green DND‐189 (Yeasen, #40767ES50). Cells were loaded with 1 µm LysoSensor in pre‐warmed medium at 37°C for 20 min. After washing with PBS, the MFI of labeled cells was detected by flow cytometry. For Lysosomal membrane potential measurement, an acridine orange staining assay was performed. Cells were incubated with 5 µg/ml acridine orange (MACKLIN, #A6009) in complete medium at 37°C for 20 min. The MFI of stained cells was measured using flow cytometry.

### 4.19 Electron Microscopy

Transmission electron microscopy was technically supported by Shanghai Jingxi Biotechnology Co., Ltd. (Shanghai, China). Cell samples were initially fixed in 2.5% glutaraldehyde at 4°C overnight, followed by post‐fixation with 1% osmium tetroxide for 2 h at room temperature and three PBS washes. After dehydration through a graded ethanol series, samples were embedded in resin, sectioned to 70 ‐ 90 nm thickness, and sequentially stained with uranyl acetate and lead citrate. Ultrastructural examination was conducted using a Hitachi HT7800 electron microscope operating at 80 kV.

### 4.20 Transwell Invasion Assay

Cell invasion capacity was assessed using Matrigel‐coated (ABclonal, #RPM0004P) transwell chambers (LABSELECT, #14341; 8 µm pore size, 6.5 mm diameter). iBMDM and HTR‐8/SVneo cells were seeded in the upper chamber, while the lower chamber contained culture medium with specified treatments. Following 24 h incubation at 37°C, non‐invading cells were removed from the upper membrane surface with a cotton swab. Invaded cells on the lower surface were fixed with 4% paraformaldehyde for 15 min, stained with 0.1% crystal violet (Servicebio, #G1014), and quantified under a microscope in five random fields.

### 4.21 Culture Media Concentration

To obtain concentrated culture supernatant, we utilized Amicon Ultra‐4 30 K centrifugal filter devices (Thermofisher, #UFC803096) with a 30 kDa molecular weight cut‐off. According to the manufacturer's protocol, 4 mL of supernatant was added to the device. Following the centrifugation at 7500 g for 30 min, molecules exceeding 30 kDa are retained with an efficiency of over 90%. After the concentration, we performed a buffer exchange into RPMI 1640 medium by adding 3.95 mL of the new medium to the filter device and repeating the centrifugation step. The resulting 50 µL concentrate was used for subsequent experiments.

### 4.22 Statistical Analysis

Statistical analysis was conducted using GraphPad Prism 10 Software. All data were presented as mean ± standard error of the mean (SEM). An unpaired, two‐tailed Student *t*‐test was used to compare the observations between two study groups. One‐way ANOVA was performed when there were multiple experimental groups. For all experiments, *p* < 0.05 was considered statistically significant. The sample size for each experiment is explicitly specified in the figure legends (e.g., n = 3 biological replicates).

## Author Contributions

L.J. and Y.Z. supervised this study. Y.Z. designed this study and drafted the manuscript. Y.Z. and H.R. edited and revised the manuscript. J.S. and Z.P. performed the animal experiments. All authors approved the final version of the manuscript.

## Conflicts of Interest

The authors declare no conflict of interest.

## Supporting information




**Supporting File**: advs73719‐sup‐0001‐SuppMat.docx.

## Data Availability

The data that support the findings of this study are available from the corresponding author upon reasonable request.
