# Supplementary material for: Perfluorooctanoic Acid Exposure Causes Macrophage Ammonia Retention and Induces Spontaneous Miscarriages
Source: Adv Sci (Weinh). 2026 Jan 4;13(16):e06994. doi: 10.1002/advs.202506994 (PMC13042366; doi:10.1002/advs.202506994)
Supplement: Supplementary file 1 — Supporting File: advs73719‐sup‐0001‐SuppMat.docx. [file ADVS-13-e06994-s001.docx]

**Perfluorooctanoic Acid Exposure Causes Macrophage Ammonia Retention and Induces Spontaneous Miscarriages**

Yongbo Zhao ^1, 2, #^, Yijun Zhang ^2, #^, Hanyu Rao ^2, #^, Jiani Sun^2, #^, Zhiyi Pan^2^, Liping Jin ^1^**^,^** ^3^**^,^** *, Yan Zhao ^1^**^,^** *

^1^ Obstetrics & Gynecology Hospital of Fudan University, Shanghai Key Lab of Reproduction and Development, Shanghai Key Lab of Female Reproductive Endocrine Related Diseases, 200433, Shanghai, China.

^2^ Shanghai Key Laboratory of Maternal Fetal Medicine, Shanghai First Maternity and Infant Hospital, School of Medicine, Tongji University, Shanghai, 201204, China

^3^ The Third Affiliated Hospital of Zhengzhou University, Zhengzhou, 450052, China

^#^ These authors contributed equally to this work

**Corresponding Authors:**

Yan Zhao and Liping Jin

Obstetrics & Gynecology Hospital of Fudan University, Shanghai Key Lab of Reproduction and Development, Shanghai Key Lab of Female Reproductive Endocrine Related Diseases, 200433, Shanghai, China.

Email: [zy861201@163.com](mailto:zy861201@163.com) (Yan Zhao); [jinlp01@163.com](mailto:jinlp01@163.com) (Liping Jin)


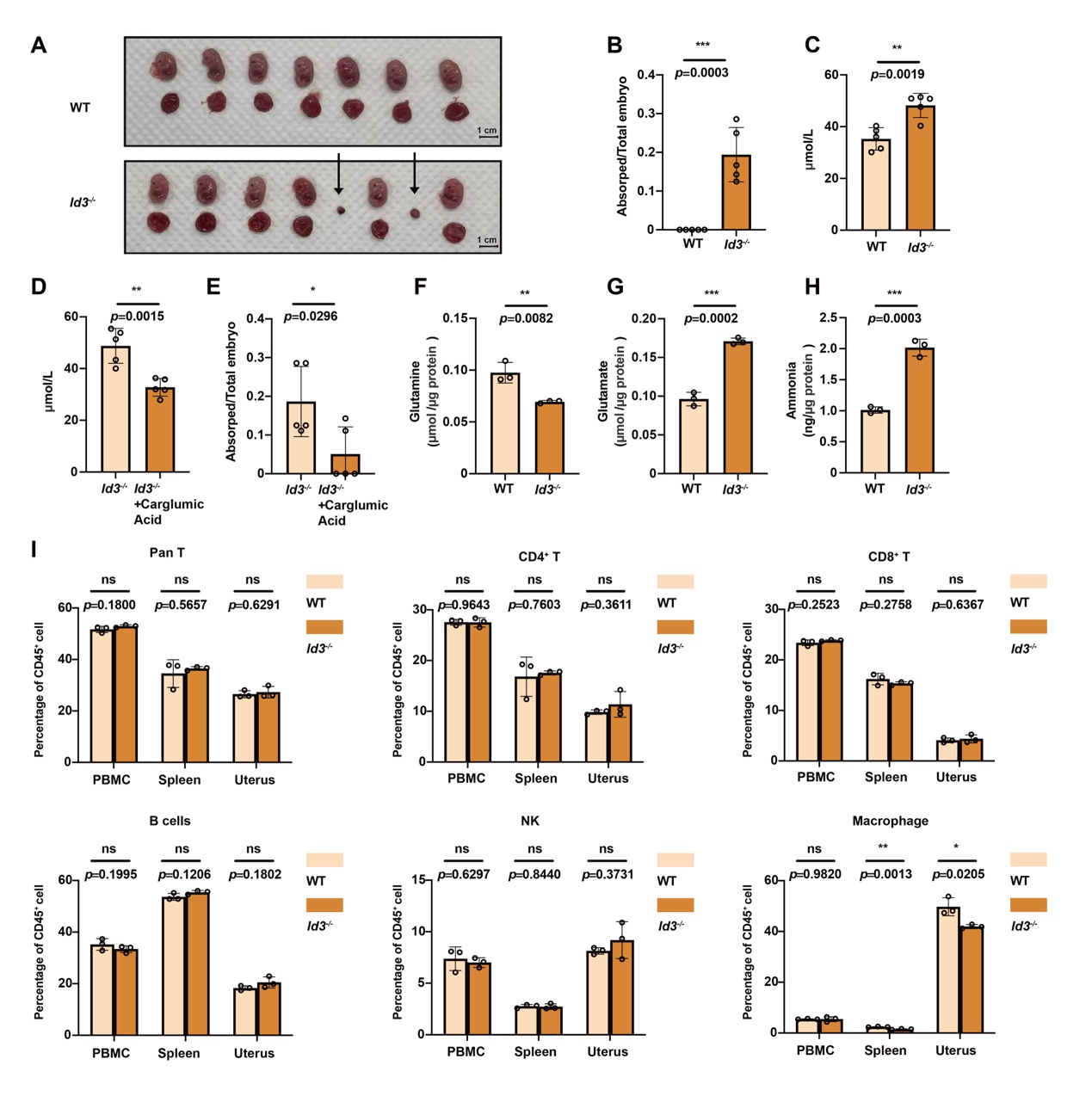


**Supplementary Figure 1. Knockout of *Id3* resulted in phenotypes similar to PFOA exposure in pregnant mice.**

(A) A representative photograph of embryos and placentas from wildtype and *Id3*^-/-^pregnant mice (arrows indicated embryo absorption site, scale bar = 1 cm). (B) Statistical analysis of the absorption rate of wildtype and *Id3*^-/-^pregnant mice (n=5). (C) Blood ammonia level in wildtype (n=5) and *Id3*^-/-^ mice (n=5). (D) Blood ammonia level in *Id3*^-/-^ mice treated with vehicle or carglumic acid (n=5). (E) Statistical analysis of the absorption rate of *Id3*^-/-^ mice treated with vehicle or carglumic acid (n=5). (F to H) Cellular concentration of glutamine, glutamate, and ammonia in BMDMs from wildtype and *Id3*^-/-^ mice (n = 3). (I) Proportions of various immune cells in the peripheral blood, spleen, and uterus of wildtype and *Id3*^-/-^ pregnant mice (n=3). Data were presented as mean ± SEM and analyzed with two-tailed unpaired Student’s *t*-test. *P* < 0.05, *; *P* < 0.01, **; *P* < 0.001, ***; *P* < 0.0001, ****; no significance, ns.


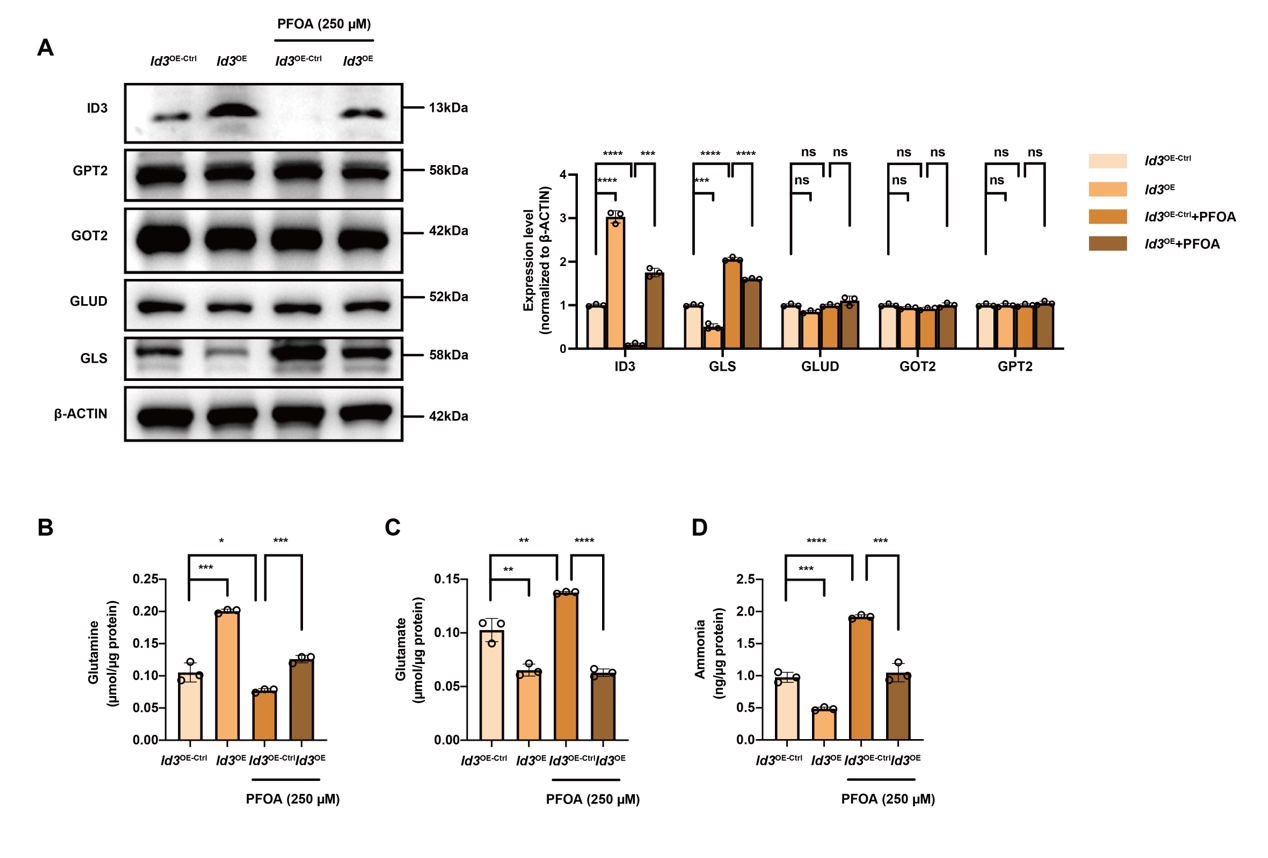


**Supplementary Figure 2. ID3 Overexpression Rescues PFOA-Induced Glutaminolysis.**

(A) Western blots of ID3, GPT2, GOT2, GLUD, and GLS in *Id3*^OE-Ctrl^, *Id3*^OE^, *Id3*^OE-Ctrl^ + PFOA, and *Id3*^OE^ + PFOA group (n = 3). The relative protein expression levels were normalized to β-ACTIN expression. (B to D) Cellular concentration of glutamine, glutamate, and ammonia in *Id3*^OE-Ctrl^, *Id3*^OE^, *Id3*^OE-Ctrl^ + PFOA, and *Id3*^OE^ + PFOA group (n = 3). Data were presented as mean ± SEM and analyzed with two-tailed unpaired Student’s *t*-test or one-way ANOVA. *P* < 0.05, *; *P* < 0.01, **; *P* < 0.001, ***; *P* < 0.0001, ****; no significance, ns.


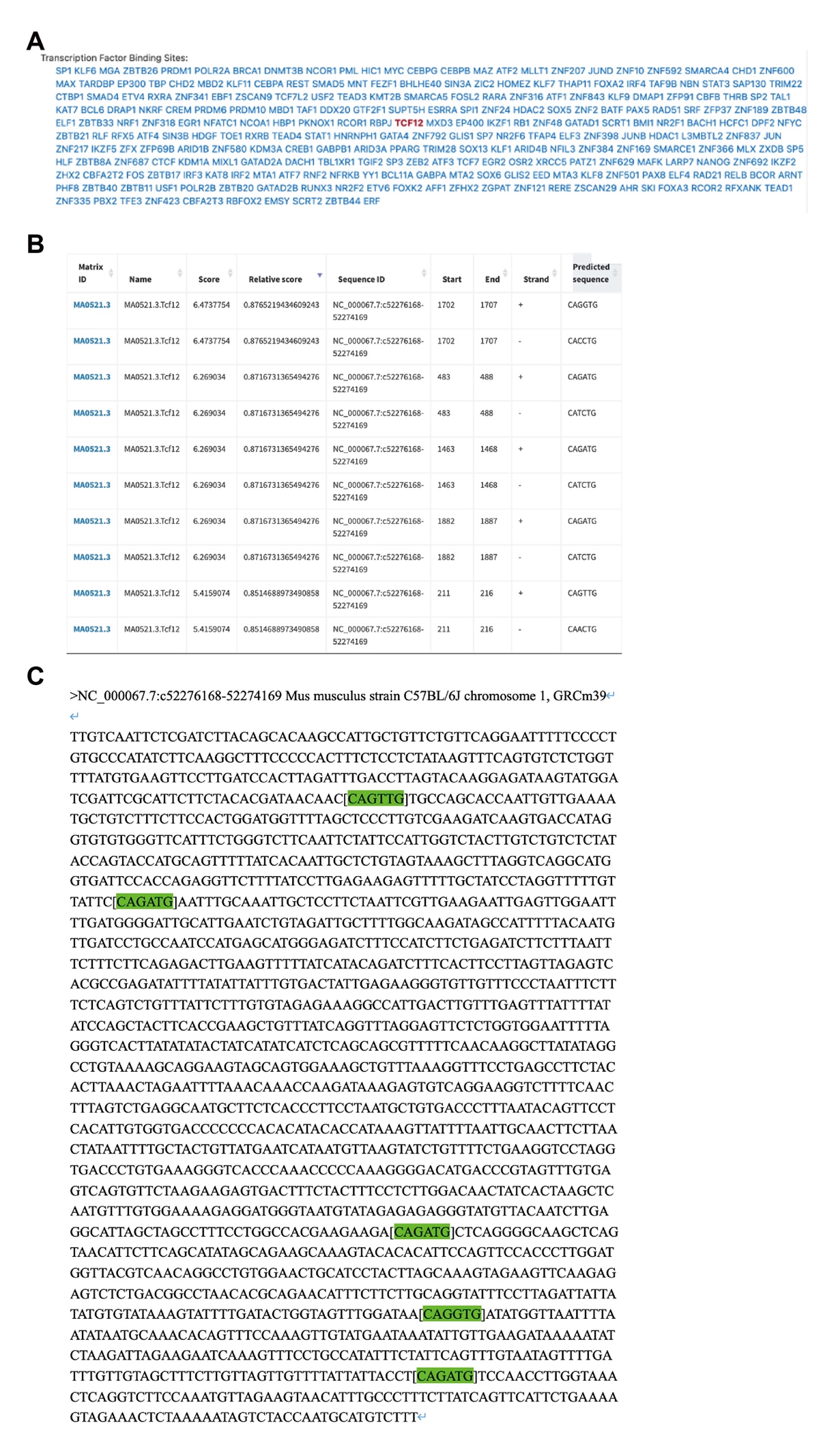


**Supplementary Figure 3. TCF12 was predicted to be the binding target of ID3.**

(A) TCF12 was predicted to interact with ID3 based on GeneCards database. (B and C) The predicted binding sites of TCF12 on the promoter region of *Gls* by the JASPAR database.


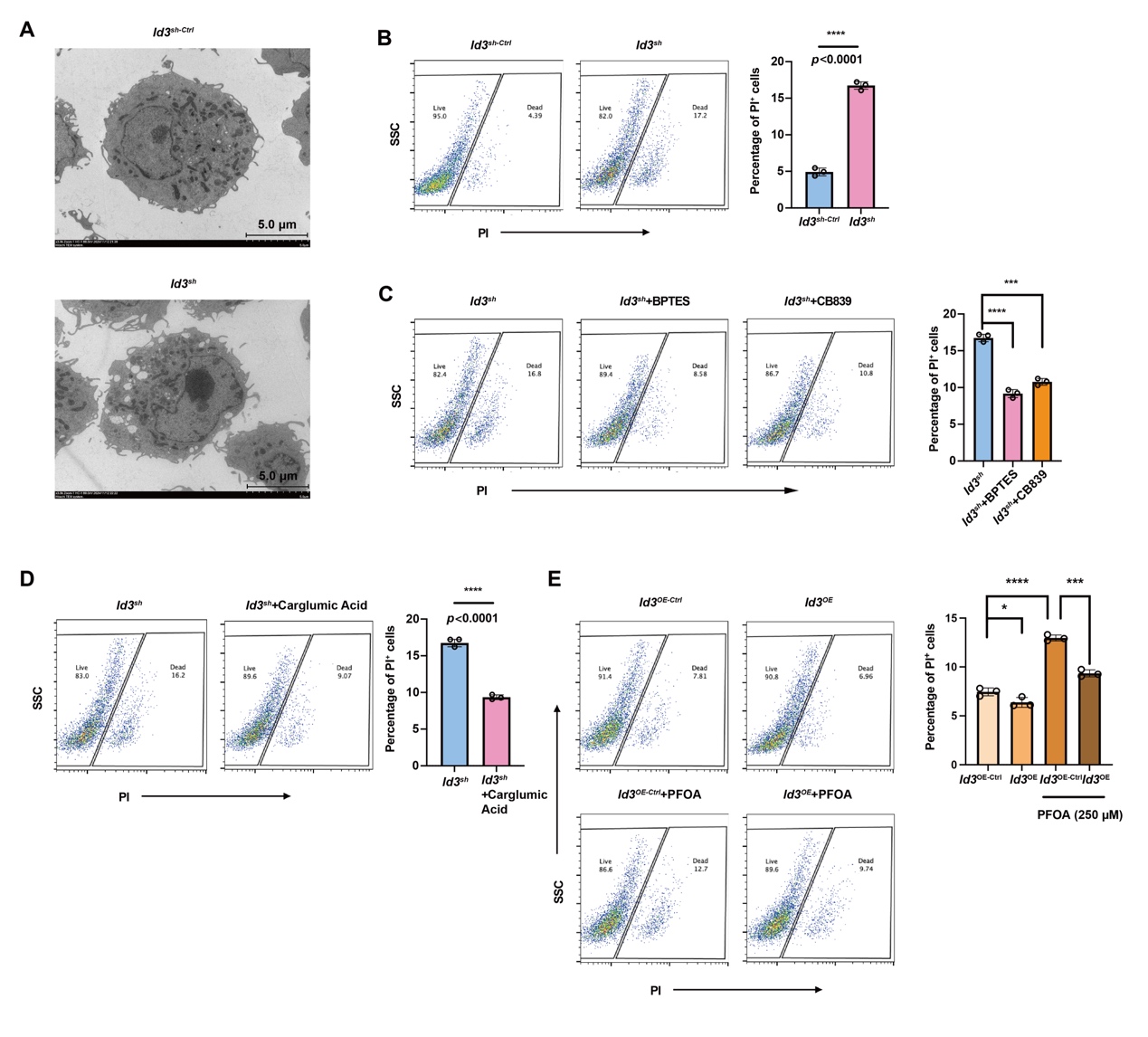
**Supplementary Figure 4. Knockdown of ID3** **decreases macrophage viability**

(A) Representative electron microscopy of sh-Ctrl together with *Id3*^sh^ macrophages. Scale bar = 500 nm. (B) PI staining of sh-Ctrl and *Id3*^sh^ macrophages (n = 3). (C) PI staining of *Id3*^sh^ macrophages, *Id3*^sh^ + BPTES group, and *Id3*^sh^ + CB839 group (n = 3). (D)PI staining of *Id3*^sh^ macrophages and *Id3*^sh^ + carglumic acid group (n = 3). (E) PI staining of *Id3*^OE-Ctrl^, *Id3*^OE^, *Id3*^OE-Ctrl^ + PFOA, and *Id3*^OE^ + PFOA group (n = 3). Data were presented as mean ± SEM and analyzed with two-tailed unpaired Student’s *t*-test or one-way ANOVA. *P* < 0.05, *; *P* < 0.01, **; *P* < 0.001, ***; *P* < 0.0001, ****; no significance, ns.


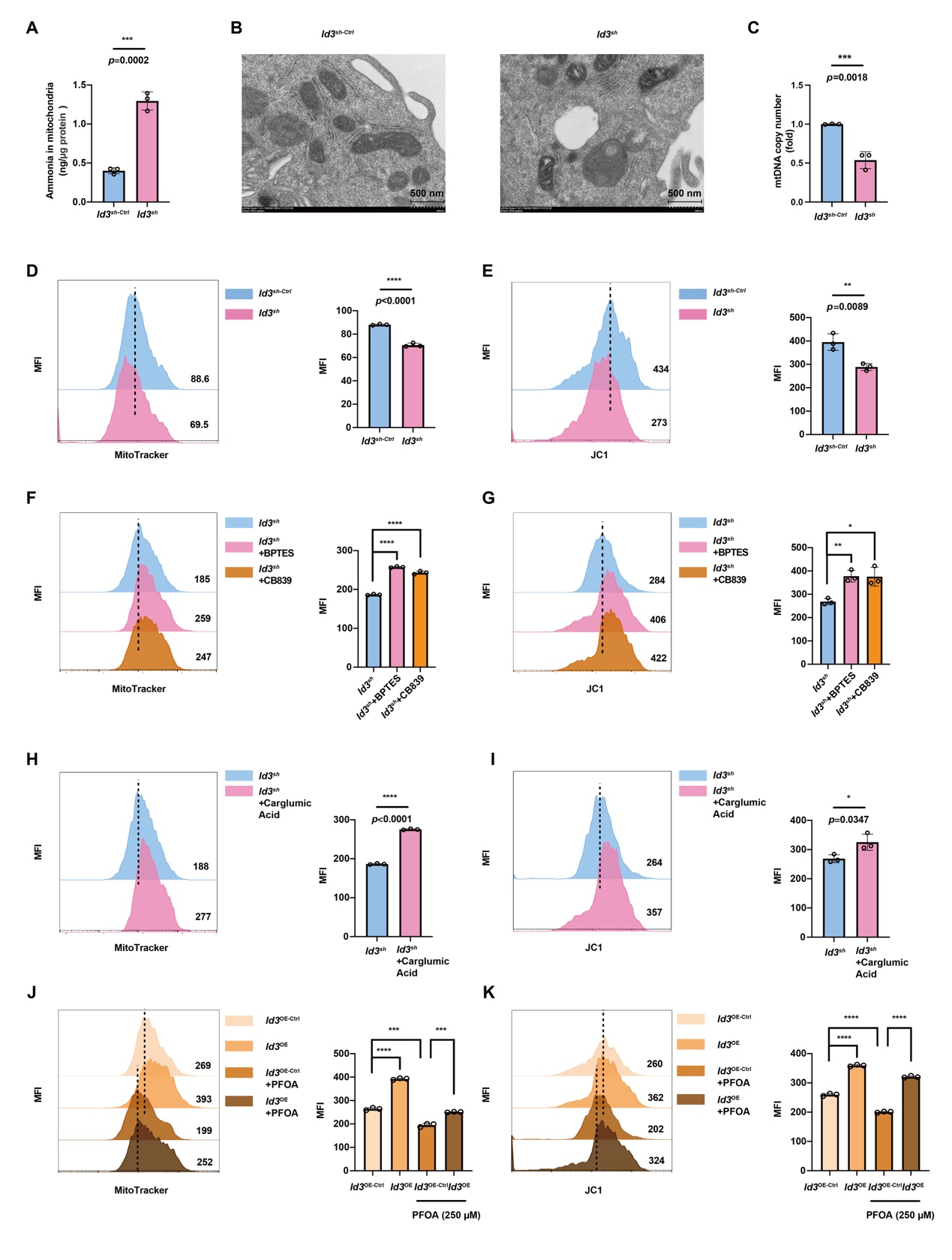


**Supplementary Figure 5. Knockdown of ID3 triggers mitochondrial dysfunction**

(A) Concentration of mitochondrial ammonia in *Id3*^sh-Ctrl^ and *Id3*^sh^ macrophages (n = 3). (B) Representative electron microscopy of mitochondria in sh-Ctrl together with *Id3*^sh^ macrophages. Scale bar = 500 nm. (C) The copy numbers of mitochondrial DNA in *Id3*^sh-Ctrl^ and *Id3*^sh^ macrophages (n = 3). (D and E) The MFI of Mito Tracker and JC-1 in *Id3*^sh-Ctrl^ and *Id3*^sh^ macrophages (n = 3). (F and G) The MFI of Mito Tracker and JC-1 in *Id3*^sh^ macrophages, *Id3*^sh^ + BPTES group, and *Id3*^sh^ + CB839 group (n = 3). (H and I) The MFI of Mito Tracker and JC-1 in *Id3*^sh^ macrophages and *Id3*^sh^ + carglumic acid group (n = 3). (J and K) The MFI of Mito Tracker and JC-1 in *Id3*^OE-Ctrl^, *Id3*^OE^, *Id3*^OE-Ctrl^ + PFOA, and *Id3*^OE^ + PFOA group (n = 3). Data were presented as mean ± SEM and analyzed with two-tailed unpaired Student’s *t*-test or one-way ANOVA. *P* < 0.05, *; *P* < 0.01, **; *P* < 0.001, ***; *P* < 0.0001, ****; no significance, ns.


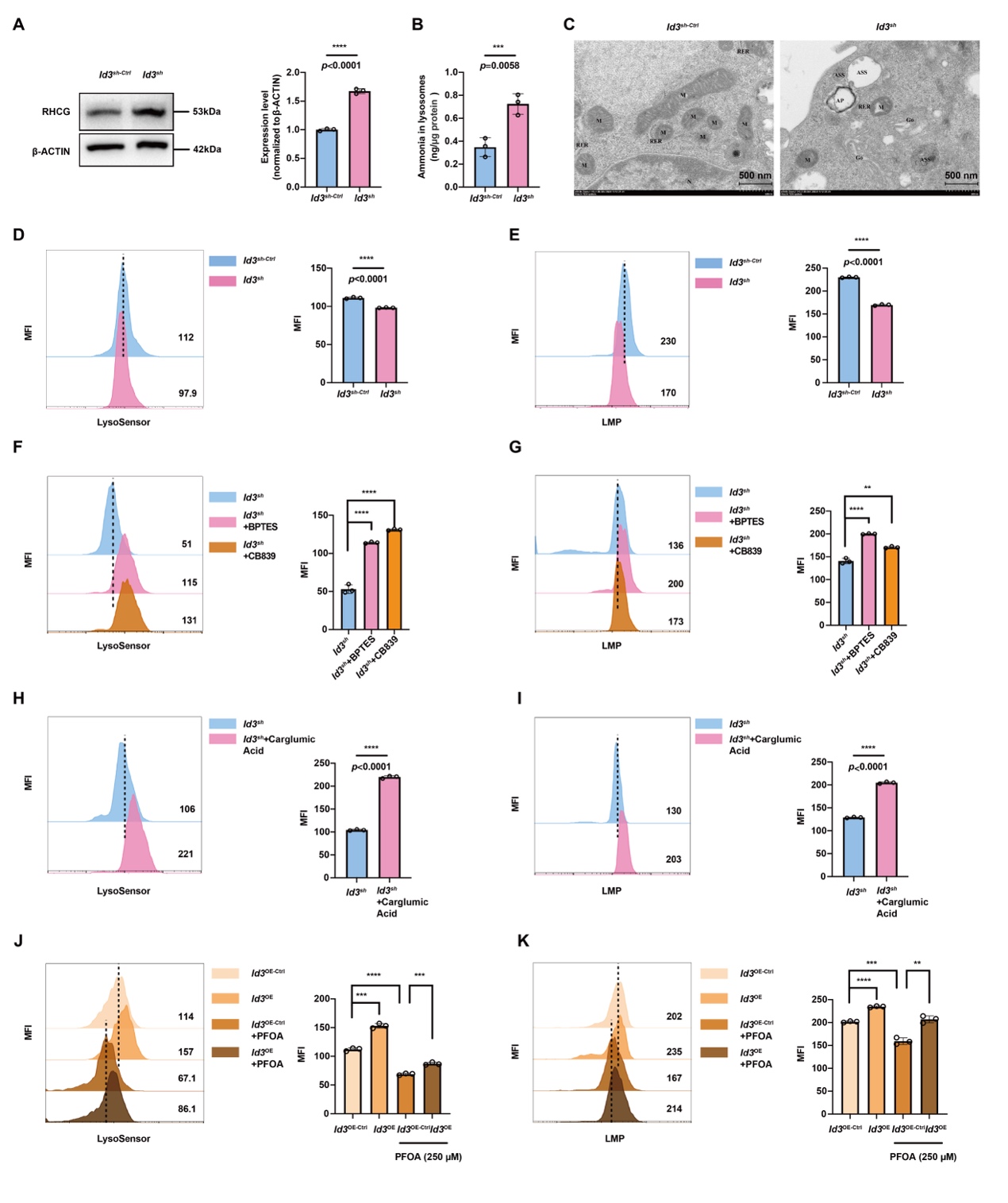


**Supplementary Figure 6. Knockdown of ID3 compromises lysosomal function**

(A) Western blots of RHCG in sh-Ctrl together with *Id3*^sh^ macrophages (n = 3). The relative protein expression levels were normalized to β-ACTIN expression. (B) Concentration of lysosomal ammonia in *Id3*^sh-Ctrl^ and *Id3*^sh^ macrophages (n = 3). (C) Representative electron microscopy of sh-Ctrl together with *Id3*^sh^ macrophages, mainly focused on autolysosomes. Scale bar = 500 nm. (D and E) The MFI of LysoSensor and acridine orange (an indicator for LMP) in *Id3*^sh-Ctrl^ and *Id3*^sh^ macrophages (n = 3). (F and G) The MFI of LysoSensor and acridine orange in *Id3*^sh^ macrophages, *Id3*^sh^ + BPTES, and *Id3*^sh^ + CB839 group (n = 3). (H and I) The MFI of LysoSensor and acridine orange in *Id3*^sh^ macrophages and *Id3*^sh^ + carglumic acid group (n = 3). (J and K) The MFI of LysoSensor and acridine orange in *Id3*^OE-Ctrl^, *Id3*^OE^, *Id3*^OE-Ctrl^ + PFOA, and *Id3*^OE^ + PFOA group (n = 3). Data were presented as mean ± SEM and analyzed with two-tailed unpaired Student’s *t*-test or one-way ANOVA. *P* < 0.05, *; *P* < 0.01, **; *P* < 0.001, ***; *P* < 0.0001, ****; no significance, ns.


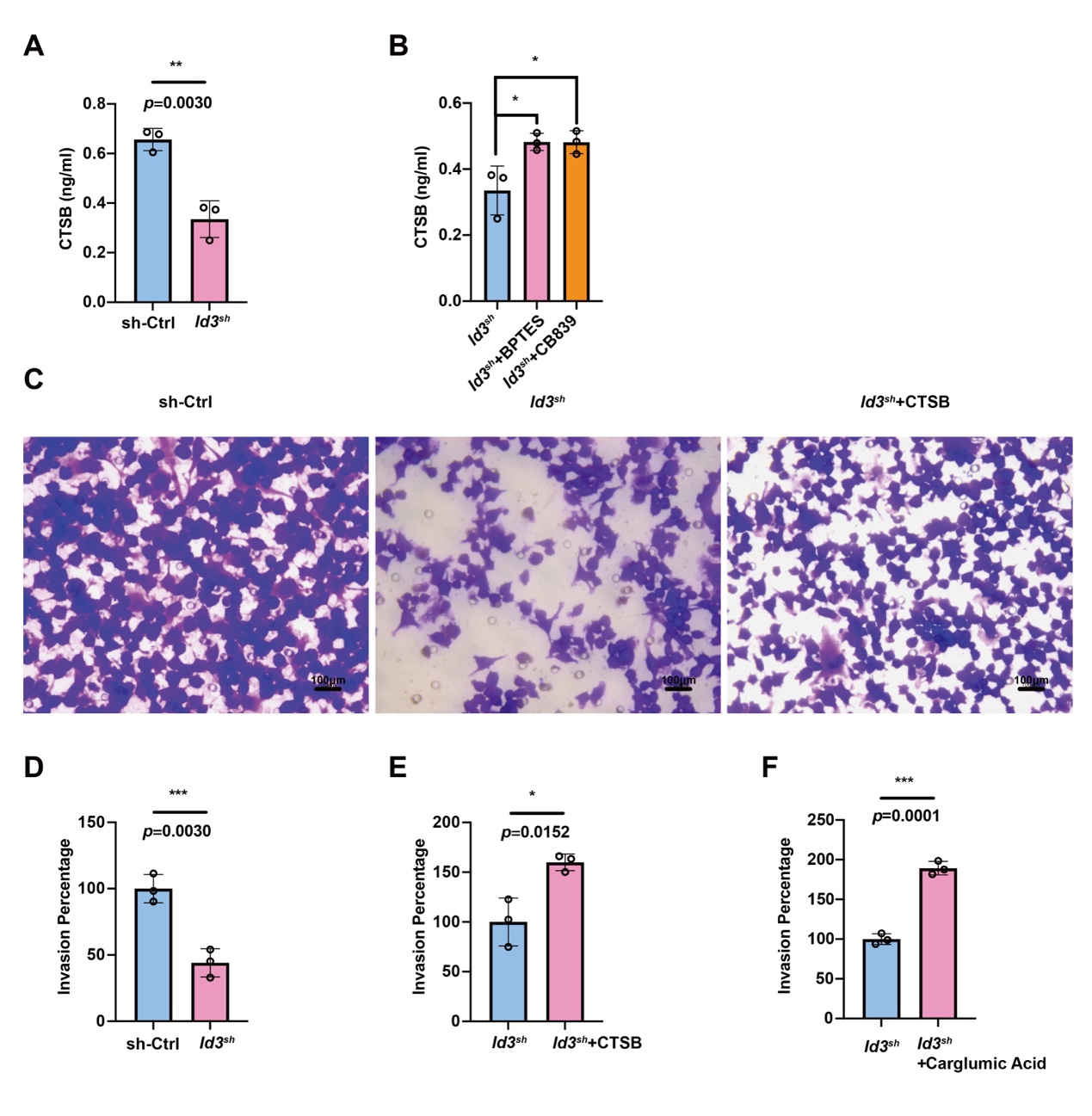
**Supplementary Figure 7. Knockdown of ID3 reduced secretion of CTSB and led to decreased macrophage infiltration**

(A) Concentration of CTSB in the culture supernatant of *Id3*^sh-Ctrl^ and *Id3*^sh^ macrophages (n = 3). (B) Concentration of CTSB in the culture supernatant of *Id3*^sh^ macrophages, *Id3*^sh^ + BPTES group, and *Id3*^sh^ + CB839 group (n = 3). (C) Representative images of *Id3*^sh^ macrophages and *Id3*^sh^ + CTSB group using transwell assay (scale bar = 100 μm). (D) Statistical analysis of the infiltration ability of *Id3*^sh-Ctrl^ and *Id3*^sh^ macrophages (n = 3). (E) Statistical analysis of the infiltration ability of *Id3*^sh^ macrophages and *Id3*^sh^ + CTSB group (n = 3). (F) Statistical analysis of the infiltration ability of *Id3*^sh^ macrophages and *Id3*^sh^ + carglumic acid group (n = 3). Data were presented as mean ± SEM and analyzed with two-tailed unpaired Student’s *t*-test or one-way ANOVA. *P* < 0.05, *; *P* < 0.01, **; *P* < 0.001, ***; *P* < 0.0001, ****; no significance, ns.


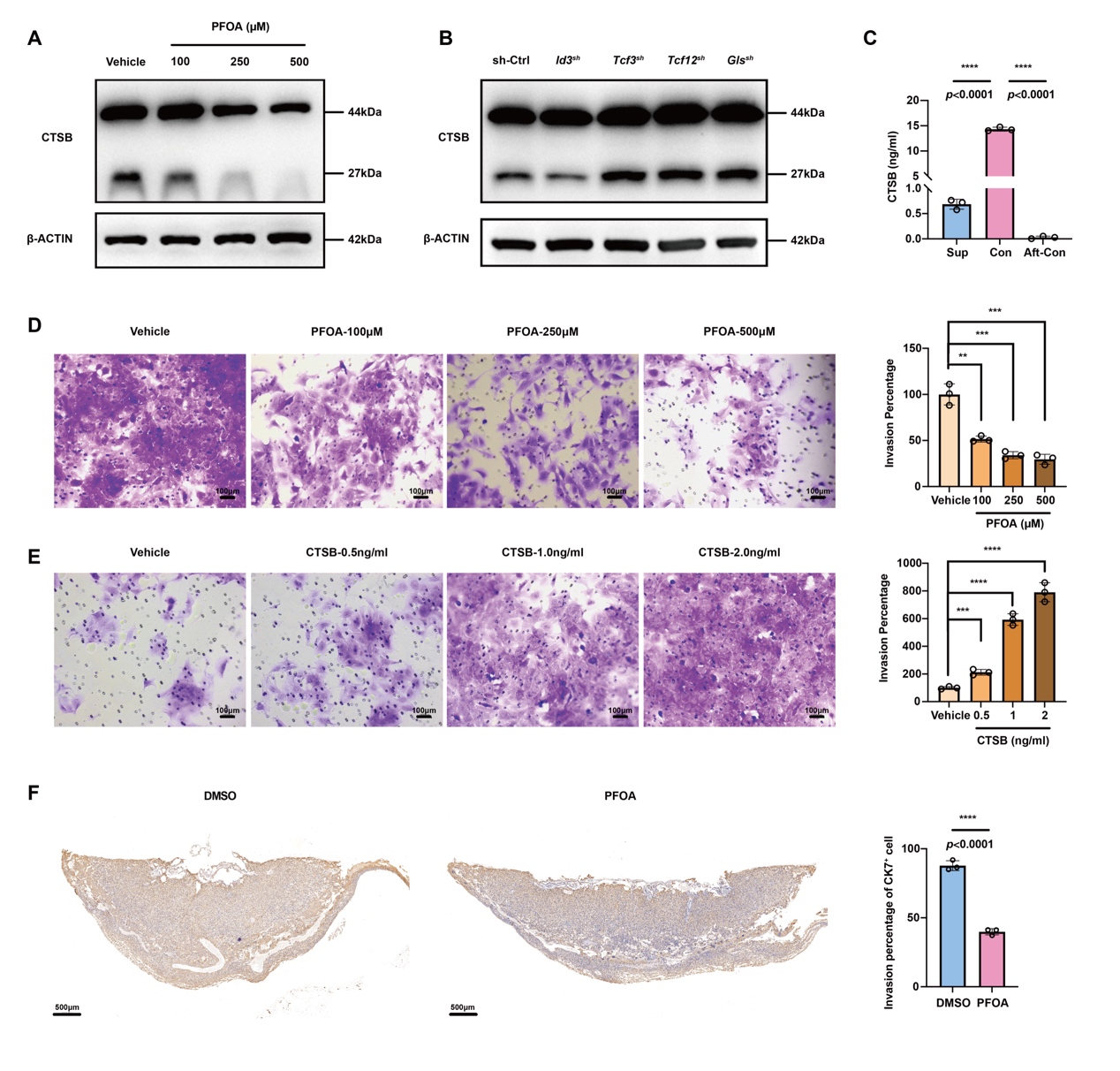
**Supplementary Figure 8. Cathepsin B facilitates trophoblast invasion.**

(A) Western blots of intracellular CTSB in macrophages treated with 0 μM, 100 μM, 250 μM, and 500 μM of PFOA. (B) Western blots of intracellular CTSB in sh-Ctrl, *Id3*^sh^, *Tcf12*^sh^, *Tcf3*^sh^, and *Gls*^sh^ macrophages. (C) Protein concentration of CTSB in the culture supernatant for PFOA-exposed macrophages, before and after protein condensation (n = 3). (Sup: supernatant for PFOA-exposed macrophages; Con: condensed supernatant; Aft-Con: fluid residual after condensation) (D) Measuring the invasion ability of trophoblasts exposed to concentrated culture supernatant of PFOA-exposed macrophages using transwell assay (scale bar = 100 μm) (n = 3). (E) Measuring the invasion ability of trophoblasts treated with exogenous CTSB using transwell assay (scale bar = 100 μm) (n = 3). (F) Measuring the invasion percentage of CK7^+^ trophoblasts in the placentas of PFOA-exposed pregnant mice by immunohistochemistry (scale bar = 500 μm) (n = 3). Data were presented as mean ± SEM and analyzed with two-tailed unpaired Student’s *t*-test or one-way ANOVA. *P* < 0.05, *; *P* < 0.01, **; *P* < 0.001, ***; *P* < 0.0001, ****; no significance, ns.

**Table S1. PFAS concentration in human decidual tissues.**

|  | **PFOA (ng/g)** | **PFNA**  **(ng/g)** | **PFDA**  **(ng/g)** | **PFHxS**  **(ng/g)** | **PFOS**  **(ng/g)** |
| --- | --- | --- | --- | --- | --- |
| **Healthy Control** |  |  |  |  |  |
| 1 | 0.894 | 0.161 | 0.162 | 0.098 | 0.689 |
| 2 | 2.08 | 0.586 | 0.911 | 0.275 | 2.38 |
| 3 | 1.56 | 0.533 | 0.699 | 0.140 | 2.21 |
| 4 | 2.61 | 0.438 | 0.430 | 0.313 | 1.65 |
| 5 | 1.24 | 0.272 | 0.312 | 0.167 | 1.49 |
| 6 | 1.52 | 0.246 | 0.291 | 0.182 | 1.74 |
| 7 | 2.05 | 0.538 | 0.489 | 0.112 | 1.86 |
| 8 | 1.89 | 0.484 | 0.484 | 0.112 | 1.98 |
| 9 | 1.51 | 0.304 | 0.362 | 0.175 | 1.53 |
| 10 | 1.66 | 0.403 | 0.433 | 0.284 | 1.70 |
| **Pregnancy Loss** |  |  |  |  |  |
| 1 | 3.55 | 0.589 | 0.812 | 0.37 | 3.38 |
| 2 | 2.76 | 0.77 | 0.702 | 0.227 | 2.14 |
| 3 | 1.47 | 0.298 | 0.365 | 0.287 | 1.36 |
| 4 | 1.78 | 0.369 | 0.461 | 0.166 | 1.46 |
| 5 | 2.63 | 0.601 | 0.999 | 0.381 | 4.85 |
| 6 | 4.56 | 1.12 | 0.586 | 0.226 | 1.69 |
| 7 | 1.54 | 0.281 | 0.367 | 0.142 | 1.21 |
| 8 | 2.52 | 0.761 | 1.10 | 0.315 | 4.63 |
| 9 | 4.52 | 0.943 | 0.663 | 0.170 | 2.42 |
| 10 | 1.39 | 0.261 | 0.268 | 0.193 | 1.23 |

**Table S2. Gene expressed level related to ID signaling pathway.**

|  | **Mean FPKM**  **of NC** | **Mean FPKM**  **of PFOA** | **log2FoldChange** | | ***p* value** |
| --- | --- | --- | --- | --- | --- |
| *Vegfa* | 154.9497 | 1861.1579 | 3.5871 | 5.1743E-128 | |
| *Lck* | 20.5551 | 60.1765 | 1.5453 | 2.3885E-09 | |
| *Acvrl1* | 440.8507 | 768.9782 | 0.8022 | 6.7906E-27 | |
| *Elk3* | 1556.5731 | 2615.6649 | 0.7487 | 3.0907E-86 | |
| *Tert* | 35.0724 | 55.0353 | 0.6511 | 5.2856E-03 | |
| *Atf3* | 3534.5944 | 4825.4288 | 0.4489 | 1.6427E-40 | |
| *Egf* | 79.3130 | 102.5641 | 0.3704 | 3.0415E-02 | |
| *Tgif1* | 1811.8413 | 2343.0253 | 0.3709 | 1.1214E-21 | |
| *Igf1r* | 444.3125 | 560.8077 | 0.3350 | 4.8564E-05 | |
| *Bmpr2* | 877.8298 | 1071.6158 | 0.2875 | 2.4474E-07 | |
| *Rb1* | 695.0558 | 835.9230 | 0.2662 | 1.8510E-04 | |
| *Elk1* | 707.7560 | 762.0364 | 0.1065 | 5.6572E-02 | |
| *Ctnnb1* | 28724.2579 | 30580.7269 | 0.0904 | 4.9988E-07 | |
| *Rbl2* | 1098.7572 | 1160.2718 | 0.0784 | 1.0986E-01 | |
| *Elk4* | 548.8683 | 574.0513 | 0.0649 | 3.4517E-01 | |
| *Rela* | 2657.8202 | 2765.0062 | 0.0567 | 1.1271E-01 | |
| *Cdk2* | 3970.5014 | 4018.8683 | 0.0173 | 5.7840E-01 | |
| *Srebf1* | 8337.2112 | 8424.4577 | 0.0150 | 6.6582E-01 | |
| *Smad4* | 2577.6843 | 2525.3743 | -0.0297 | 3.3951E-01 | |
| *Tcf12* | 4054.4790 | 3896.3894 | -0.0572 | 5.5137E-02 | |
| *Tcf3* | 5019.3201 | 4812.5727 | -0.0608 | 1.6060E-01 | |
| *Smad3* | 807.7794 | 761.3935 | -0.0852 | 1.5019E-01 | |
| *Smad5* | 1662.4853 | 1542.4147 | -0.1081 | 7.8776E-03 | |
| *Tcf7l2* | 427.5797 | 373.3600 | -0.1957 | 1.8386E-02 | |
| *Bmp2* | 1316.4149 | 1480.3793 | 0.1692 | 3.0352E-04 | |
| *Rbl1* | 1851.9919 | 1504.9869 | -0.2994 | 1.7575E-10 | |
| *Psmd4* | 9810.5487 | 7774.3998 | -0.3356 | 7.1178E-25 | |
| *Nfkb1* | 16999.7006 | 13139.4084 | -0.3716 | 9.1547E-77 | |
| *Smad1* | 415.2520 | 307.4286 | -0.4332 | 2.2843E-07 | |
| *Flt1* | 6631.6579 | 4811.3507 | -0.4629 | 1.4407E-48 | |
| *Igf1* | 10247.0090 | 6347.0103 | -0.6910 | 2.5288E-128 | |
| *Ccna2* | 8377.6123 | 4200.9409 | -0.9959 | 0.0000E+00 | |
| *Ccne1* | 2515.1239 | 1246.4949 | -1.0128 | 5.3373E-118 | |
| *Id1* | 1967.4701 | 295.4777 | -2.7354 | 3.2115E-298 | |
| *Id2* | 777.3115 | 76.9939 | -3.3359 | 2.0218E-224 | |
| *Id3* | 8530.2558 | 257.7875 | -5.0487 | 0.0000E+00 | |

**Table S3. Primers used for mouse genotyping.**

|  | **Forward Primer (5' - 3')** | **Reverse Primer (5' - 3')** |
| --- | --- | --- |
| Pair1 | CTGGTCCACACTCCTTGTCC | GCCACCCAAGTTCAGTCCTT |
| Pair2 | CCGTGGTATCTGGGTTTGCT | GCAGCCACGAGATTGCTTTC |

**Table S4. Antibodies used in flow cytometry.**

| **Antibody** | **Source** | | **Catalogue** |
| --- | --- | --- | --- |
| Brilliant Violet 421™ anti-mouse CD45 | | BioLegend | 103134 |
| FITC anti-mouse CD11b | | BioLegend | 101206 |
| PE/Cyanine7 anti-mouse F4/80 | | BioLegend | 123114 |
| FITC anti-mouse CD3 | | BioLegend | 100203 |
| PerCP/Cyanine5.5 anti-mouse CD4 | | BioLegend | 100434 |
| APC anti-mouse CD8a | | BioLegend | 100712 |
| PE anti-mouse NK-1.1 | | BioLegend | 108707 |
| PE/Cyanine7 anti-mouse CD19 | | BioLegend | 115519 |
| Propidium Iodide Solution | | BioLegend | 421301 |

**Table S5. Primers used to construct vectors.**

| **Name** | **Sequence (5' - 3')** |
| --- | --- |
| *Id3* F | CCGGGCAGCGTGTCATAGACTACATCTCGAGATGTAGTCTATGACACGCTGCTTTTTG |
| *Id3* R | AATTCAAAAAGCAGCGTGTCATAGACTACATCTCGAGATGTAGTCTATGACACGCTGC |
| *Tcf12* F | CCGGTGACGATTTCAACCGTGAATCCTCGAGGATTCACGGTTGAAATCGTCATTTTTG |
| *Tcf12* R | AATTCAAAAATGACGATTTCAACCGTGAATCCTCGAGGATTCACGGTTGAAATCGTCA |
| *Tcf3* F | CCGGGCACATCGTGCCTAAGCATTTCTCGAGAAATGCTTAGGCACGATGTGCTTTTTG |
| *Tcf3* R | AATTCAAAAAGCACATCGTGCCTAAGCATTTCTCGAGAAATGCTTAGGCACGATGTGC |
| *Gls* F | CCGGAGAAAGTGGAGATCGAAATTTCTCGAGAAATTTCGATCTCCACTTTCTTTTTTG |
| *Gls* R | AATTCAAAAAAGAAAGTGGAGATCGAAATTTCTCGAGAAATTTCGATCTCCACTTTCT |

**Table S6. Antibodies used for Western blot.**

| **Antibody** | **Source** | **Catalogue** | **Dilution** |
| --- | --- | --- | --- |
| anti-β-Actin | ABclonal | AC038 | 1:5000 |
| anti-GPT2 | ABclonal | A23670 | 1:1000 |
| anti-GOT2 | ABclonal | A19245 | 1:1000 |
| anti-GLUD1 | ABclonal | A5176 | 1:1000 |
| anti-GLS | ABclonal | A23189 | 1:1000 |
| anti-ID3 | ABclonal | A5375 | 1:500 |
| anti-TCF3 | ABclonal | A23946 | 1:1000 |
| anti-TCF12 | ABclonal | A4146 | 1:1000 |
| anti-GAPDH | ABclonal | AC001 | 1:3000 |
| anti-Lamin A/C | ABclonal | A0249 | 1:3000 |
| anti-RHCG | ABclonal | A16124 | 1:1000 |
| anti-Cathepsin B | ABclonal | A0967 | 1:1000 |

**Table S7. Primers used in qRT-PCR assay.**

|  | **Forward Primer (5' - 3')** | **Reverse Primer (5' - 3')** |
| --- | --- | --- |
| *Actb* | GGCTGTATTCCCCTCCATCG | CCAGTTGGTAACAATGCCATGT |
| *Gls* | GACAACGTCAGATGGTGTCAT | TGCTTGTGTCAACAAAACAATGT |
| *Id3* | CGACCGAGGAGCCTCTTAG | GGACGCGATAGGGAAGACC |
| *NucActb* | CTAAGGCCAACCGTGAAAAG | ACCAGAGGCATACAGGGACA |
| *Mito12S* | ACCGCGGTCATACGATTAAC | CCCAGTTTGGGTCTTAGCTG |

**Table S8. Primers used in ChIP assay.**

|  | **Forward Primer (5' - 3')** | **Reverse Primer (5' - 3')** |
| --- | --- | --- |
| *Gls-211* | TGGATCGATTCGCATTCTTCT | CCCAGAAATGAACCCACACA |
| *Gls-483* | AGGTCAGGCATGGTGATTCC | TCCCCATCAAAATTCCAACTCA |
| *Gls-1463* | TGTTTGTGGAAAAGAGGATGGG | ATGTTACTGAGCTTGCCCCT |
| *Gls-1702* | GGCCTAACACGCAGAACATT | ACTTTGGAAACTGTGTTTGCA |
| *Gls-1882* | TGCAAACACAGTTTCCAAAGT | GGAAGACCTGAGTTTACCAAGG |
